# The quasi-xgamma frailty model with survival analysis under heterogeneity problem, validation testing, and risk analysis for emergency care data

**DOI:** 10.1038/s41598-024-59137-w

**Published:** 2024-04-18

**Authors:** Hamami Loubna, Hafida Goual, Fatimah M. Alghamdi, Manahil SidAhmed Mustafa, Getachew Tekle Mekiso, M. Masoom Ali, Abdullah H. Al-Nefaie, Hassan Alsuhabi, Mohamed Ibrahim, Haitham M. Yousof

**Affiliations:** 1 Laboratory of Probabilities and Statistics LaPS, Department of Mathematics, Faculty of Sciences, Badji Mokhtar Annaba University, Annaba, Algeria; 2 Department of Mathematical Sciences, College of Science, Princess Nourah bint Abdulrahman University, P.O. Box 84428, 11671 Riyadh, Saudi Arabia; 3 Department of Statistics, Faculty of Science, University of Tabuk, Tabuk, Saudi Arabia; 4 Department of Statistics, College of Natural and Computational Science, Wachemo University, Hossana, Ethiopia; 5 Department of Mathematical Sciences, Ball State University, Muncie, IN USA; 6 Department of Quantitative Methods, School of Business, King Faisal University, 31982 Al-Ahsa, Saudi Arabia; 7 Department of Mathematics, Al-Qunfudah University College, Umm Al-Qura University, Mecca, Saudi Arabia; 8 Department of Applied, Mathematical and Actuarial Statistics, Faculty of Commerce, Damietta University, Damietta, Egypt; 9 Department of Statistics, Mathematics and Insurance, Benha University, Benha, Egypt

**Keywords:** Censored data, Frailty model, Heterogeneity, Maximum likelihood, Statistical test, Survival analysis, Statistics, Applied mathematics

## Abstract

Frailty models are important for survival data because they allow for the possibility of unobserved heterogeneity problem. The problem of heterogeneity can be existed due to a variety of factors, such as genetic predisposition, environmental factors, or lifestyle choices. Frailty models can help to identify these factors and to better understand their impact on survival. In this study, we suggest a novel quasi xgamma frailty (QXg-F) model for the survival analysis. In this work, the test of Rao–Robson and Nikulin is employed to test the validity and suitability of the probabilistic model, we examine the distribution’s properties and evaluate its performance in comparison with many relevant cox-frailty models. To show how well the QXg-F model captures heterogeneity and enhances model fit, we use simulation studies and real data applications, including a fresh dataset gathered from an emergency hospital in Algeria. According to our research, the QXg-F model is a viable replacement for the current frailty modeling distributions and has the potential to improve the precision of survival analyses in a number of different sectors, including emergency care. Moreover, testing the ability and the importance of the new QXg-F model in insurance is investigated using simulations via different methods and application to insurance data.

## Introduction

Survival analysis is an important statistical tool that is used to investigate time-to-event data. One example of this type of data is the period of time that passes between the diagnosis of an illness and the occurrence of an interesting event, such as death or recurrence. Survival analysis is extensively utilized in a wide variety of fields, including medicine, biology, economics, engineering, and the social sciences. In survival analysis, one of the most important assumptions to make is that the time-to-event data follow an independent and identical (IID) distribution. This assumption is not always accurate, and the data are vulnerable to unobserved heterogeneity or fragility, both of which have the potential to have an effect on a person’s chance of surviving. Shared frailty models and independent frailty models are the two primary categories of frailty models that may be found in the medical literature. Shared frailty models operate under the presumption that all persons within a cluster have the same type of vulnerability, whereas independent frailty models operate under the presumption that each person has their own particular vulnerability. The usage of shared frailty models is common in situations in which the individuals who make up a cluster are linked to one another, for as in the case of twins or members of the same family. Independent frailty models are often utilized in situations in which the people who make up a cluster are not connected to one another in any way, such as patients who are participating in a therapeutic trial. Estimating the hazard-rate function, which is the chance of an occurrence occurring at a particular period, can be done with the use of fragility models. Predicting an individual’s or a group’s chance of survival by using the hazard-rate function is possible. This can be done for an individual or for a group of persons. It is also possible to utilize frailty models to determine which factors are related with an increased or decreased risk of an occurrence. (for further reading, see^1–3^).

In frailty modelling, the most fundamental premise is that the observed data are produced by a mix of independent and identically distributed random variables (IIRRV) and a random frailty factor that reflects the unobserved heterogeneity in the data. This is known as the independent and IIRRV model. According to Aalen and Tretli^4^, frailty modelling has been shown to be a useful method for evaluating survival data in a variety of settings, including cancer research, clinical trials, and epidemiology. The analysis of survival data can be greatly aided by the use of frailty models. They can be used to predict the probability of survival for an individual or a group of persons, and they can assist in the identification of characteristics that are related with an increased or decreased risk of an occurrence. Numerous distributions, such as the gamma distribution^1,5^, the compound Poisson distribution^6,7^, the log-normal distribution^8^. Pickles and Crouchley^9^ examined the effectiveness of conditional and mixture likelihood approaches in estimating models incorporating frailty effects in censored bivariate survival data. Their study revealed that mixture methods exhibit remarkable resilience to frailty distribution misspecification. Additionally, the paper includes an illustrative example involving the onset times of chest pain induced by three endurance exercise tests during a drug treatment trial involving heart patients. Therneau and colleagues (2002) showed that exact solutions for gamma shared frailty models can be achieved through penalized estimation. Likewise, Gaussian frailty models are closely associated with penalized models. Efficient fitting of frailty models with penalized likelihoods can be facilitated by leveraging computational techniques available for penalized models. We have incorporated penalized regression into the coxph function of S-Plus and demonstrate the algorithms with examples employing the Cox model. Box–Steffensmeier and De Boef^10^ conducted a comparison of different models for analyzing recurrent event data characterized by both heterogeneity and event dependence. They found that the conditional frailty model offers the most comprehensive approach to addressing the diverse conditions of heterogeneity and event dependence, utilizing a frailty term, stratification, and gap time formulation of the risk set. The study evaluates the effectiveness of recurrent event models frequently employed in practical applications through Monte Carlo simulations, and applies the insights gained to data concerning chronic granulomatous disease and cystic fibrosis.

Recently, Jiang and Haneuse^11^ introduced a novel class of transformation models tailored for semi-competing risks analysis, allowing for the non-parametric specification of the frailty distribution. The weighted Lindley frailty (see^12^), have been suggested in the statistical and reliability literature to explain the frailty term. However, the ability of these distributions to truly represent the variability of the data is limited due to the constraints they impose.

It has been demonstrated that the new QXg-F model is a suitable replacement for the gamma frailty model, the compound Poisson frailty model, the log-normal frailty model, and the weighted Lindley frailty model. It is important to note that the new frailty model is derived based on the quasi Xgamma (QXg) model that was initially presented by Sen and Chandra (2017). This is something that should be mentioned. The proposed QXg-F model is able to take into account unobserved variability, which helps to improve the fit of the frailty model. The suggested distribution is based on the QXg-F model, which has been shown to have good flexibility in modelling a wide range of data types. This flexibility has been proved through modelling. We extend the QXg-F model by incorporating a fragility component and show that the resulting distribution possesses desirable features such as positive support, skewness, and kurtosis. The Nikulin Rao and Robson (NIK-RR) test is a modified version of the chi-squared goodness-of-fit test that was proposed by Nikulin^13^, Nikulin^14^, Nikulin^15^, Nikulin^13^, and Rao and Robson^16^ for completed data. This test was used to validate the proposed QXg-F model. In the event where censored data are present, an adjusted version of the chi-squared goodness-of-fit test known as the Bagdonavicius–Nikulin (B-NIK) test was developed by Bagdonavicius and Nikulin^17^. This test was used to validate the suggested QXg-F model. It is worth noting that the literature (statistical and actuarial) contains many important extensions and various applications of the x-gamma distribution, see for example^18–20^

It is worth mentioning that, both the NIK-RR test statistic and the B-NIK test statistic are statistical tests used to assess the goodness of fit of a distribution to a set of data. However, there are some important differences between the two tests:

- The NIK-RR test statistic is a general test of goodness of fit, meaning it can be used to test the goodness of fit to any distribution. On the other hand, the B-NIK test statistic is specifically designed to test the goodness of fit to the normal distribution.
- The NIK-RR test statistic is based on the comparison of the ECDF to a reference distribution, while the B-NIK test statistic is based on the comparison of the sample mean and variance to their expected values under the assumption of normality.
- The NIK-RR test statistic has been shown to be more powerful than the B-NIK test statistic in some situations, especially when the sample size is small or when the data is not exactly normal.

For the purpose of this investigation, we gathered fresh, actual data from an Algerian emergency room, and we referred to this information as emergency care data. We model the time-to-event data for the individuals in the sample who have a given medical condition by utilising methodologies from survival analysis. The newly collected data on emergency care is analyzed using the recommended QXg-F model, the frailty model under the Weibull baseline hazard-rate function (WBH-F) (also known as the Weibull frailty (W-F) model), and the Gompertz baseline hazard-rate function (GBH-F) (also known as the Gompertz frailty (G-F) model. We show that the QXg-F model that was recommended is a good fit for the new data on emergency care, and that it produces correct estimates of the survival function and the hazard rate. Therefore, the QXg-F model demonstrated its superiority when compared to both the W-F model and the G-F model. Furthermore, in the field of analysing and evaluating the risks that insurance companies are exposed to by evaluating and analysing insurance claims data by studying a set of commonly used financial indicators such as the value-at-risk (V-R), tail-value-at-risk (TLV-R), tail variance (T-VC), tail Mean-Variance (TM-V), and the mean excess loss (M-EXL) function (see Furman The following methods of estimation are addressed for the purpose of computing the primary key risk indicators (KRIs): the maximal likelihood estimate, also known as maximum likelihood estimation (MaxLE), the ordinary least squares estimation, also known as OrLSE, the weighted least squares estimation, and the Anderson Darling estimation, also known as AnDE. These four aforementioned approaches were used and applied in two different directions of financial and actuarial assessment, one of which was simulation under three confidence levels (C-Ls), and various sample sizes are considered for applications to insurance claims data. The other route involved the use of these methods in a different manner.

The main motivation of this paper is to:

- Present a new flexible frailty model called the QXg-F model for the survival analysis.
- Employ the QXg-F model in the survival analysis under a newly collected data called emergency care data.
- Using the MaxLE method is used for estimating the QXg-F model’s parameters of WBH-F and in case of GBH-F.
- Propose an alternative frailty model which overcomes the weak point of the gamma frailty model.
- Testing the validity using the NIK-RR test statistic in case of complete data.
- Testing the validity using the B-NIK test statistic in case of censored data.
- Test the ability of the new QXg-F model in risk analysis by studying a set of commonly used financial indicators such as the V-R, TLV-R, T-VC, TM-V, M-EXL function under different estimation methods like the MaxLE method, OrLSE method, the WLSE method, and the AnDE method.

In short, the primary focus of this study is the introduction of the QXg-F Model. This model expands upon the traditional gamma frailty model by using a more adaptable distribution, namely the Xgamma distribution. By doing this, it offers a more adaptable method for modelling the vulnerability impact in the context of emergency care data. This model enables a more precise depiction of the unobserved variability among individuals, a common occurrence in healthcare data sets. The research utilizes the QXg-F Model to analyze a dataset from the emergency care area. The conducted survival study within the model yields valuable data regarding the probability of survival and rates of hazard for patients. It aids in identifying the key elements that have a major impact on survival outcomes in this particular scenario. The authors perform thorough validation tests to evaluate the performance of the QXg-F Model. They utilize goodness-of-fit tests, cross-validation, and other validation approaches to guarantee the accuracy and dependability of the model. The results of these tests establish confidence in the model’s ability to capture the underlying heterogeneity in the data.

The paper expands its analysis to include risk assessment, enabling a thorough review of patient outcomes in the emergency care context. The practical ramifications of this research are highly relevant for healthcare practitioners, as it can provide valuable insights for making decisions regarding the allocation of resources, treatment procedures, and patient prioritization. The QXg-F Model is a new and inventive statistical technique. This tool enhances the selection of frailty models for survival analysis, therefore serving as a vital asset in the statistical toolkit for researchers across many disciplines. By utilizing the model on emergency care data, the research establishes a connection between theoretical statistical technique and practical healthcare applications. This study offers valuable information on survival outcomes and risk factors that can inform clinical decision-making. The model used in this study has undergone comprehensive validation testing, ensuring its reliability and robustness. Researchers can be certain of its relevance to other datasets exhibiting comparable properties. The results of this study have significant ramifications for both the scientific community and medical professionals. The QXg-F Model holds promise as a valuable instrument for analyzing healthcare data, specifically in the realm of emergency care. The capacity to consider diversity and precisely calculate survival probabilities can aid healthcare practitioners in making well-informed decisions about patient care and resource distribution. Moreover, the paper’s focus on validation and testing establishes a benchmark for statistical modelling in the healthcare field. Implementing this methodology in additional research endeavours can bolster the dependability of findings and guarantee that statistical models faithfully depict the fundamental facts.

## The Cox-frailty model

Consider the Cox proportional hazard (Cox-PH) model (see^21^) and an unexplained source of heterogeneity. Let $Z>0$ and for an unobserved random variable that represents the frailty of the object. Then, the hazard-rate function for the i^th^ item is

1 $$\begin{aligned} \lambda \left( t_{i}|z_{i},x_{i}\right) =z_{i}\lambda _{0}(t_{i})\exp (x_{i}^{T}\underline{\varvec{\beta }})|i=1,2,...,n, \end{aligned}$$

where $\lambda _{0}(\cdot )$ refers to the hazard-rate function of the baseline model, $\underline{\varvec{\beta }}=\underline{ \varvec{\beta }}_{\left( p\times 1\right) }$ is the vector of unknown regression coefficients for all $p<n$ (see^22^), where subject *i* has a unique frailty $z_{i}$, which is an unobserved non-negative number. Hence, if $z_{i}>1$ or $z_{i}<1$, respectively, frailty $z_{i}$ raises or reduces the chance of occurrence of the event of our interest. The Cox-PH model is produced as a specific instance where $z_{i}=1$ for every *i*. The following is how the model in Eq. (1) is used to determine the conditional survival function for the *i*th subject:

2 $$\begin{aligned} S(t_{i}|z_{i},x_{i})=\exp (-z_{i}\Lambda _{0}(t_{i})\exp (x_{i}^{T} \underline{\varvec{\beta }}))|i=1,.....,n \end{aligned}$$

where the cumulative baseline hazard-rate function is $\Lambda _{0}(t_{i})=\int _{0}^{t_{i}}\lambda _{0}(s)ds$. The conditional survival function (2) thus indicates the likelihood that the i^th^ subject will live until time $t_{i}$ given $Z=z_{i}$. We must integrate out the conditional survival function (Eq. 2) on frailty in order to obtain the marginal survival (Mar-S) function, which does not depend on unseen variables. Keep in mind that this is equal to computing the frailty distribution’s Laplace transform. In reality, if *f*(*z*) is the frailty distribution, then we may get the following by integrating $S(t_{i}\mid z_{i},x_{i})$ from Eq. (2) on $Z=z_{i}$:

3 $$\begin{aligned} S(t_{i}|x_{i})=\int _{0}^{\infty }\exp (-z_{i}\Lambda _{0}(t_{i})\exp (x_{i}^{T}\underline{\varvec{\beta }}))f(z_{i})dz_{i}=L_{f}(\Lambda _{0}(t_{i})\exp (x_{i}^{T}\underline{\varvec{\beta }})) \end{aligned}$$

where $L_{f}(\cdot )$ stands for the frailty distribution’s Laplace transform, and the appropriate marginal probability density function (MPDF) is

$$\begin{aligned} f(t_{i}|x_{i})=-\lambda _{0}(t_{i})\exp (x_{i}^{T}\underline{\varvec{\beta }} )L_{f}^{^{\prime }}\left[ \Lambda _{0}(t_{i})\exp (x_{i}^{T}\underline{ \varvec{\beta }})\right] |i=1,.....,n. \end{aligned}$$

Take into account that the Laplace transform has a closed form for each of the distributions above. As a result, Eq. (3) may be used to derive the marginal hazard (Mar-H) function as follows:

4 $$\begin{aligned} \lambda (t_{i}|x_{i})=-\frac{\lambda _{0}(t_{i})\exp (x_{i}^{T}\underline{ \varvec{\beta }})L_{f}^{^{\prime }}\left[ \Lambda _{0}(t_{i})\exp (x_{i}^{T} \underline{\varvec{\beta }})\right] }{L_{f}(\Lambda _{0}(t_{i})\exp (x_{i}^{T}\underline{\varvec{\beta }}))}|i=1,2,...,n. \end{aligned}$$

where $L_{f}^{^{\prime }}(t)=\frac{\partial }{\partial t}L_{f}(t)$. The risk and survival of a person randomly selected from the research population are therefore calculated using the hazard and Mar-S functions (illustrated above) (see^23^). If the frailty distribution does not have a Laplace transform in closed form, it becomes necessary to use numerical integration or Markov Chain Monte Carlo methods (see^24–27^). The consideration of frailty distribution is crucial for ensuring computational simplicity in both univariate and multivariate survival data modeling (see^9,23^). Hazard and Mar-S functions were created in this study.

### The QXg-F model

Following Sen and Chandra (2017), the probability density function (PDF) of the QXg model can be expressed as

5 $$\begin{aligned} f_{\underline{\textbf{P}}}(z)=\frac{\theta }{1+\zeta }\exp \left( -\theta z\right) \left( \zeta +\frac{1}{2}\theta ^{2}z^{2}\right) |\zeta ,\theta >0, \end{aligned}$$

where $\underline{\textbf{P}}=\left( \zeta ,\theta \right) .$ Consider the frailty model in Eq. (1) where *Z* is our frailty variable which has a QXg distribution with mean one. This assumption is required to identify the parameters of the derived model (see^28^). Hence, employing Mazucheli’s^29^ alternate parameterization of the QXg model in terms of mean, the PDF of the QXg-F model becomes

6 $$\begin{aligned} f(Z)=\frac{3+\zeta }{\left( 1+\zeta \right) ^{2}}\exp \left( -\frac{3+\zeta }{1+\zeta }z\right) \left( \zeta +\frac{1}{2}\left( \frac{3+\zeta }{1+\zeta } \right) ^{2}z^{2}\right) \end{aligned}$$

where $\zeta >0$ represents the (unknown) shape parameter. The variance of the frailty distribution is commonly used to quantify the level of unobserved variation in a research sample. If the PDF (5) is assumed to be our frailty model, then, the variance can be expressed as

$$\begin{aligned} \sigma ^{2}=\frac{1}{(3+\zeta )^{2}}\left( \zeta ^{2}+8\zeta +3\right) . \end{aligned}$$

The frailty PDF (6)’s Laplace transform, depending on its variance and $S\in {\mathbb {R}}$, is given by:

7 $$\begin{aligned} L_{f}(S)= &{} \left( 1+\sqrt{{\mathbb {C}}\left[ \sigma ^{2}\right] }\right) \nonumber \\{} & {} \times \left[ \begin{array}{c} \left( 4-3\sigma ^{2}+\sqrt{{\mathbb {C}}\left[ \sigma ^{2}\right] }\right) \\ \left( S(3-2\sigma ^{2}+\sqrt{{\mathbb {C}}\left[ \sigma ^{2}\right] })+1+\sqrt{ {\mathbb {C}}\left[ \sigma ^{2}\right] }\right) ^{2}+\left( 1+\sqrt{{\mathbb {C}} \left[ \sigma ^{2}\right] }\right) ^{2}(\sigma ^{2}-1) \end{array} \right] \nonumber \\ &\times \left\{ (3-2\sigma ^{2}+\sqrt{{\mathbb {C}}\left[ \sigma ^{2}\right] } )\left( S(3-2\sigma ^{2}+\sqrt{{\mathbb {C}}\left[ \sigma ^{2}\right] })+1+ \sqrt{{\mathbb {C}}\left[ \sigma ^{2}\right] }\right) ^{3}\right\} ^{-1}. \end{aligned}$$

where ${\mathbb {C}}\left[ \sigma ^{2}\right] =13-12\sigma ^{2}$ and$\ \sigma ^{2}\le \frac{13}{12}.$ In order to maintain simplicity, we analyze (7) at $S=\Lambda _{0}(t_{i})\xi _{i}$, where $\xi _{i}$ =$\exp (x_{i}^{T}\underline{ \varvec{\beta }})$, and find that the Mar-S function (Eq. 3) under QXg-F model is provided by

8 $$\begin{aligned} S(t_{i}|x_{i})= & {} \left( 1+\sqrt{{\mathbb {C}}\left[ \sigma ^{2}\right] }\right) \nonumber \\{} & {} \times \left[ \begin{array}{c} \left( 4-3\sigma ^{2}+\sqrt{{\mathbb {C}}\left[ \sigma ^{2}\right] }\right) \\ \left( \begin{array}{c} \Lambda _{0}(t_{i})\exp (x_{i}^{T}\underline{\varvec{\beta }})(3-2\sigma ^{2}+\sqrt{{\mathbb {C}}\left[ \sigma ^{2}\right] }) \\ +1+\sqrt{{\mathbb {C}}\left[ \sigma ^{2}\right] } \end{array} \right) ^{2} \\ +\left( 1+\sqrt{{\mathbb {C}}\left[ \sigma ^{2}\right] }\right) ^{2}(\sigma ^{2}-1) \end{array} \right] \nonumber \\{} & {} \times \left\{ \begin{array}{c} (3-2\sigma ^{2}+\sqrt{{\mathbb {C}}\left[ \sigma ^{2}\right] }) \\ \left( \begin{array}{c} \Lambda _{0}(t_{i})\exp (x_{i}^{T}\underline{\varvec{\beta }}) \\ \times (3-2\sigma ^{2}+\sqrt{{\mathbb {C}}\left[ \sigma ^{2}\right] })+1+\sqrt{ {\mathbb {C}}\left[ \sigma ^{2}\right] } \end{array} \right) ^{3} \end{array} \right\} ^{-1}. \end{aligned}$$

The resulting Mar-H function (Eq. 4) is as follows:

9 $$\begin{aligned} \lambda (t_{i}|x_{i})= & {} \left[ \lambda _{0}(t_{i})\exp (x_{i}^{T}\underline{ \varvec{\beta }})\right] \nonumber \\{} & {} \left[ \frac{(1+\zeta )}{\left( \Lambda _{0}(t_{i})\exp (x_{i}^{T} \underline{\varvec{\beta }})(1+\zeta )+3+\zeta \right) }\right] \nonumber \\{} & {} \left[ \frac{\zeta \left[ \Lambda _{0}(t_{i})\exp (x_{i}^{T}\underline{ \varvec{\beta }})(1+\zeta )+3+\zeta \right] ^{2}+3\left( 3+\zeta \right) ^{2} }{\zeta \left[ \Lambda _{0}(t_{i})\exp (x_{i}^{T}\underline{\varvec{\beta }} )(1+\zeta )+3+\zeta \right] ^{2}+\left( 3+\zeta \right) ^{2}}\right] , \end{aligned}$$

where

$$\begin{aligned} \zeta= & {} \frac{1}{\sigma ^{2}-1}\left( 4-3\sigma ^{2}+\sqrt{{\mathbb {C}}\left[ \sigma ^{2}\right] }\right) |\sigma ^{2}\ge \frac{13}{12},\\ 1+\zeta= & {} \frac{1}{\sigma ^{2}-1}\left( 3-2\sigma ^{2}+\sqrt{{\mathbb {C}}\left[ \sigma ^{2}\right] }\right) |\sigma ^{2}\ge \frac{13}{12}, \end{aligned}$$

and

$$\begin{aligned} 3+\zeta =\frac{1}{\sigma ^{2}-1}\left( 1+\sqrt{{\mathbb {C}}\left[ \sigma ^{2} \right] }\right) |\sigma ^{2}\ge \frac{13}{12}. \end{aligned}$$

### The QXg-F model under the WBH-F

Consider the baseline W model, then have:

10 $$\begin{aligned} \lambda _{0}(t_{i})=\ \frac{\kappa }{\rho }\left( \frac{t_{i}}{\rho } \right) ^{\kappa -1}\ \ \text {and\ }\ \ \Lambda _{0}(t_{i})=\left( \frac{ t_{i}}{\rho }\right) ^{\kappa }{{\vert }}t_{i}>0 \end{aligned}$$

where $\kappa >0$ represents the shape parameter and $\rho >0$ represents the scale parameter. The hazard-rate function of the Weibull distribution drops monotonously for $\kappa <1$; it is constant over time for $\kappa =1$ (exponential distribution); and it grows monotony when $\kappa >1$^23^. As a result of plugging Eq. (10) into Eqs. (9) and (8), the QXg-F model’s Mar-S and hazard functions with WBH-F are, respectively,

11 $$\begin{aligned} S(t_{i}|x_{i})= & {} \left( 1+\sqrt{{\mathbb {C}}\left[ \sigma ^{2}\right] }\right) \nonumber \\{} & {} \times \left[ \begin{array}{c} \left( 4-3\sigma ^{2}+\sqrt{{\mathbb {C}}\left[ \sigma ^{2}\right] }\right) \\ \left( \begin{array}{c} \left( \frac{t_{i}}{\rho }\right) ^{\kappa }\text { }\exp (x_{i}^{T} \underline{\varvec{\beta }}) \\ (3-2\sigma ^{2}+\sqrt{{\mathbb {C}}\left[ \sigma ^{2}\right] })+1+\sqrt{\mathbb { C}\left[ \sigma ^{2}\right] } \end{array} \right) ^{2} \\ +\left( 1+\sqrt{{\mathbb {C}}\left[ \sigma ^{2}\right] }\right) ^{2}(\sigma ^{2}-1) \end{array} \right] \nonumber \\ \times{} & {} \left\{ \begin{array}{c} (3-2\sigma ^{2}+\sqrt{{\mathbb {C}}\left[ \sigma ^{2}\right] }) \\ \left( \begin{array}{c} \left( \frac{t_{i}}{\rho }\right) ^{\kappa }\text { }\exp (x_{i}^{T} \underline{\varvec{\beta }}) \\ \times (3-2\sigma ^{2}+\sqrt{{\mathbb {C}}\left[ \sigma ^{2}\right] })+1+\sqrt{ {\mathbb {C}}\left[ \sigma ^{2}\right] } \end{array} \right) ^{3} \end{array} \right\} ^{-1} \end{aligned}$$

and

12 $$\begin{aligned} \lambda (t_{i}|x_{i})= & {} \left[ \frac{\kappa }{\rho }\left( \frac{t_{i}}{ \rho }\right) ^{\kappa -1}\exp (x_{i}^{T}\underline{\varvec{\beta }})\right] \nonumber \\{} & {} \times \left[ \frac{3-2\sigma ^{2}+\sqrt{{\mathbb {C}}\left[ \sigma ^{2} \right] }}{\left( \left( \frac{t_{i}}{\rho }\right) ^{\kappa }\exp (x_{i}^{T} \underline{\varvec{\beta }})(3-2\sigma ^{2}+\sqrt{{\mathbb {C}}\left[ \sigma ^{2}\right] })+1+\sqrt{{\mathbb {C}}\left[ \sigma ^{2}\right] }\right) }\right] \nonumber \\{} & {} \times \left[ \frac{ \begin{array}{c} 4-3\sigma ^{2}+\sqrt{{\mathbb {C}}\left[ \sigma ^{2}\right] }\left( \begin{array}{c} \left( \frac{t_{i}}{\rho }\right) ^{\kappa }\exp (x_{i}^{T}\underline{ \varvec{\beta }})(3-2\sigma ^{2} \\ +\sqrt{{\mathbb {C}}\left[ \sigma ^{2}\right] })+1+\sqrt{{\mathbb {C}}\left[ \sigma ^{2}\right] } \end{array} \right) ^{2} \\ +3(\sigma ^{2}-1)\left( 1+\sqrt{{\mathbb {C}}\left[ \sigma ^{2}\right] }\right) ^{2} \end{array} }{ \begin{array}{c} 4-3\sigma ^{2}+\sqrt{{\mathbb {C}}\left[ \sigma ^{2}\right] }\left( \begin{array}{c} \left( \frac{t_{i}}{\rho }\right) ^{\kappa }\exp (x_{i}^{T}\underline{ \varvec{\beta }})(3-2\sigma ^{2} \\ +\sqrt{{\mathbb {C}}\left[ \sigma ^{2}\right] }) \\ +1+\sqrt{{\mathbb {C}}\left[ \sigma ^{2}\right] } \end{array} \right) ^{2} \\ +(\sigma ^{2}-1)\left( 1+\sqrt{{\mathbb {C}}\left[ \sigma ^{2}\right] }\right) ^{2} \end{array} }\right] \end{aligned}$$

### The QXg-F model under the GBH-F

Consider the baseline G model, then have:

13 $$\begin{aligned} \lambda _{0}(t_{i})=\rho _{1}\exp (\kappa _{1}t_{i})\ \ \ \ \text {and\ }\ \ \ \Lambda _{0}(t_{i})=\frac{\rho _{1}}{\kappa _{1}}(\exp (\kappa _{1}t_{i})-1)\ \ \ \ \ ,t_{i}>0 \end{aligned}$$

where $\kappa _{1}>0$ and $\rho _{1}>0$ are the shape and scale parameters. If $\kappa _{1}<0$, the Gompertz distribution is flawed (Calsavara et al. (2019a) and Calsavara et al. (2019b)), since its cumulative hazard-rate function converges to the constant $-\dfrac{\rho _{1}}{\kappa _{1}}$ for $t\rightarrow \infty$, resulting in a cure or long-term survivors fraction $p_{0}=exp(\frac{\rho _{1}}{\kappa _{1}})$ in the research population. The exponential distribution is derived as a special case for $\kappa _{1}=0$. As a result, the Gompertz distribution’s hazard-rate function might be decreasing $(\kappa _{1}<0)$, constant $(\kappa _{1}=0)$, or increasing $(\kappa _{1}>0)$. The Mg-Su and hazard functions of the QXg-F model with GBH-F are calculated by inserting Eq. (12) into Eqs. (9) and (8),

14 $$\begin{aligned} S(t_{i}|x_{i})= & {} \left( 1+\sqrt{{\mathbb {C}}\left[ \sigma ^{2}\right] }\right) \nonumber \\{} & {} \times \left[ \begin{array}{c} \left( 4-3\sigma ^{2}+\sqrt{{\mathbb {C}}\left[ \sigma ^{2}\right] }\right) \\ \left( \begin{array}{c} \frac{\rho _{1}}{\kappa _{1}}(\exp (\kappa _{1}t_{i})-1)\exp (x_{i}^{T} \underline{\varvec{\beta }}) \\ \times (3-2\sigma ^{2}+\sqrt{{\mathbb {C}}\left[ \sigma ^{2}\right] })+1+\sqrt{ {\mathbb {C}}\left[ \sigma ^{2}\right] } \end{array} \right) ^{2} \\ +\left( 1+\sqrt{{\mathbb {C}}\left[ \sigma ^{2}\right] }\right) ^{2}(\sigma ^{2}-1) \end{array} \right] \nonumber \\{} & {} \times \left\{ \begin{array}{c} \left( 3-2\sigma ^{2}+\sqrt{{\mathbb {C}}\left[ \sigma ^{2}\right] }\right) \\ \times \left( \begin{array}{c} \frac{\rho _{1}}{\kappa _{1}}(\exp (\kappa _{1}t_{i})-1)\exp (x_{i}^{T} \underline{\varvec{\beta }})(3-2\sigma ^{2} \\ +\sqrt{{\mathbb {C}}\left[ \sigma ^{2}\right] })+1+\sqrt{{\mathbb {C}}\left[ \sigma ^{2}\right] } \end{array} \right) ^{3} \end{array} \right\} ^{-1} \end{aligned}$$

and

$$\begin{aligned} \lambda (t_{i}|x_{i})= & {} \left[ \rho _{1}\exp (\kappa _{1}t_{i})\exp (x_{i}^{T}\underline{\varvec{\beta }})\right] \\{} & {} \times \left[ \frac{3-2\sigma ^{2}+\sqrt{{\mathbb {C}}\left[ \sigma ^{2} \right] }}{\left( \frac{\rho _{1}}{\kappa _{1}}(\exp (\kappa _{1}t_{i})-1)\exp (x_{i}^{T}\underline{\varvec{\beta }})(3-2\sigma ^{2}+ \sqrt{{\mathbb {C}}\left[ \sigma ^{2}\right] })+1+\sqrt{{\mathbb {C}}\left[ \sigma ^{2}\right] }\right) }\right] \\{} & {} \times \left[ \frac{ \begin{array}{c} 4-3\sigma ^{2}+\sqrt{{\mathbb {C}}\left[ \sigma ^{2}\right] }\left( \begin{array}{c} \frac{\rho _{1}}{\kappa _{1}}(\exp (\kappa _{1}t_{i})-1)\exp (x_{i}^{T} \underline{\varvec{\beta }}) \\ (3-2\sigma ^{2}+\sqrt{{\mathbb {C}}\left[ \sigma ^{2}\right] })+1+\sqrt{\mathbb { C}\left[ \sigma ^{2}\right] } \end{array} \right) ^{2} \\ +3(\sigma ^{2}-1)\left( 1+\sqrt{{\mathbb {C}}\left[ \sigma ^{2}\right] }\right) ^{2} \end{array} }{ \begin{array}{c} 4-3\sigma ^{2}+\sqrt{{\mathbb {C}}\left[ \sigma ^{2}\right] }\left( \begin{array}{c} \frac{\rho _{1}}{\kappa _{1}}(\exp (\kappa _{1}t_{i})-1) \\ \exp (x_{i}^{T}\underline{\varvec{\beta }})(3-2\sigma ^{2}+\sqrt{{\mathbb {C}} \left[ \sigma ^{2}\right] }) \\ +1+\sqrt{{\mathbb {C}}\left[ \sigma ^{2}\right] } \end{array} \right) ^{2} \\ +(\sigma ^{2}-1)\left( 1+\sqrt{{\mathbb {C}}\left[ \sigma ^{2}\right] }\right) ^{2} \end{array} }\right] \end{aligned}$$

## Estimating the QXg-F model

### Case of WBH-F

The MxLEs have appealing qualities under specific regularity constraints, such as consistency, efficiency, asymptotic normality, and others^30^. It is conceivable that lifetime data will not be accessible for all research participants. Certain lives, for example, are right-censored and are merely known to be greater than the recorded figure. If so, let $T_{i}$ and $C_{i}$ be the lifespan and censoring time variables for the *i*th person in the population under investigation, respectively. Assume $T_{i}$ and $C_{i}$ are independent random variables, and $\delta _{i}=\textbf{I}_{(T_{i}\le C_{i})}$ is the censoring indicator (i.e., $\delta _{i}=1$ if $T_{i}$ is lifetime, and $\delta _{i}=0$ otherwise). Then we see that $t_{i}=min\{T_{i},C_{i}\}$. Let $x_{i}$ represent a $p\times 1$ vector of variables observed in the $i^{th}$ subject. The likelihood function for the model parameter vector $\underline{\textbf{P}}$ under non-informative censoring is thus provided from a sample of *n* participants as $L(\underline{\textbf{P}})=\overset{n}{\underset{i=1}{\prod }}\lambda (t_{i}\mid x_{i})^{\delta _{i}}S(t_{i}\mid x_{i}),$ where $S(\cdot \mid x_{i})$ and $\lambda (\cdot \mid x_{i})$ are the Mg-Su and hazard functions given in Eqs. (8) and (9). As a result, the associated log-likelihood function is calculated using the natural logarithm of $L(\underline{\textbf{P}})$. Then, the log likelihood function for $\underline{\textbf{P}}=(\kappa ,\rho ,\sigma ^{2},\underline{ \varvec{\beta }})$ is given by

15 $$\begin{aligned} \log L(\underline{\textbf{P}})= & {} r\log (\kappa )+(\kappa -1)\overset{n}{ \underset{i=1}{\sum }\delta _{i}}\log (t_{i})-Kr\log (\rho )+\overset{n}{ \underset{i=1}{\sum }}\delta _{i}x_{i}^{T}\underline{\varvec{\beta }} \nonumber \\{} & {} +\overset{n}{\underset{i=1}{\sum }}\delta _{i}\log (3-2\sigma ^{2}+\sqrt{ {\mathbb {C}}\left[ \sigma ^{2}\right] })-\overset{n}{\underset{i=1}{\sum }} \delta _{i}\log \left( A_{1}\right) \nonumber \\{} & {} +\overset{n}{\underset{i=1}{\sum }}\delta _{i}\log \left( B_{1}\right) -\sum \delta _{i}\log \left( C_{1}\right) +\underset{i=1}{\overset{n}{\sum }}\log (1+\sqrt{{\mathbb {C}}\left[ \sigma ^{2}\right] }) \nonumber \\{} & {} +\underset{i=1}{\overset{n}{\sum }}\log \left( C_{1}\right) -\underset{i=1 }{\overset{n}{\sum }}\log (3-2\sigma ^{2}+\sqrt{{\mathbb {C}}\left[ \sigma ^{2} \right] })-\underset{i=1}{\overset{n}{\sum }}3\log \left( A_{1}\right) ; \end{aligned}$$

where: $r=\overset{n}{\underset{i=1}{\sum }}\delta _{i}$ is the failure number,

$$\begin{aligned} A_{1}= & {} \left( \dfrac{t_{i}}{\rho }\right) ^{\kappa }\exp (x_{i}^{T}\underline{ \varvec{\beta }})(3-2\sigma ^{2}+\sqrt{{\mathbb {C}}\left[ \sigma ^{2}\right] } )+1+\sqrt{{\mathbb {C}}\left[ \sigma ^{2}\right] },\\ B_{1}= & {} \left[ \begin{array}{c} \left( 4-3\sigma ^{2}+\sqrt{{\mathbb {C}}\left[ \sigma ^{2}\right] }\right) \\ \times \left( \begin{array}{c} \left( \frac{t_{i}}{\rho }\right) ^{\kappa }\exp (x_{i}^{T}\underline{ \varvec{\beta }})(3-2\sigma ^{2} \\ +\sqrt{{\mathbb {C}}\left[ \sigma ^{2}\right] }) \\ +1+\sqrt{{\mathbb {C}}\left[ \sigma ^{2}\right] } \end{array} \right) ^{2} \end{array} \right] +3(1+\sqrt{{\mathbb {C}}\left[ \sigma ^{2}\right] })^{2}(\sigma ^{2}-1) \end{aligned}$$

and

$$\begin{aligned} C_{1}=\left[ \begin{array}{c} \left( 4-3\sigma ^{2}+\sqrt{{\mathbb {C}}\left[ \sigma ^{2}\right] }\right) \\ \times \left( \begin{array}{c} \left( \frac{t_{i}}{\rho }\right) ^{\kappa }\exp (x_{i}^{T}\underline{ \varvec{\beta }})(3-2\sigma ^{2} \\ +\sqrt{{\mathbb {C}}\left[ \sigma ^{2}\right] }) \\ +1+\sqrt{{\mathbb {C}}\left[ \sigma ^{2}\right] } \end{array} \right) ^{2} \end{array} \right] +(1+\sqrt{{\mathbb {C}}\left[ \sigma ^{2}\right] })^{2}(\sigma ^{2}-1). \end{aligned}$$

Setting the nonlinear system of the score equations $\textbf{I}_{\left( \kappa \right) }=0,\textbf{I}_{\left( \rho \right) }=0,\textbf{I}_{\left( \sigma ^{2}\right) }=0$ and $\textbf{I}_{\left( \underline{\varvec{\beta }} \right) }=0$ and solving them simultaneously yields the MaxLE $\widehat{ \underline{\textbf{P}}}=(\widehat{\kappa },\widehat{\rho },\widehat{\sigma ^{2}},\widehat{\underline{\varvec{\beta }}})^{\intercal }$. It is usually more convenient to use nonlinear optimization methods to solve these equations; such as the quasi-Newton algorithm to numerically maximize $\log L(\underline{\textbf{P}})$, for more applications see^30^.

### Simulations: case of WBH-F

We consider the QXg-F model with WBH-F. The data were simulated $N=13,000$ times; with parameter values $\kappa =0.8,$ $\rho =0.9,$ $\sigma ^{2}=0.6,$ $\beta _{1}=0.5$ and sample sizes $n=20,$ $n=50,$ $n=300$ and $n=1000$. Due to Ravi and Gelpert^31^, the BB (Barzilai-Borwein) algorithm is considered for estimating the average values of the MaxLE. $\widehat{\kappa },\widehat{\rho },\widehat{\sigma ^{2}},\widehat{ \beta _{1}}$ parameters and their mean squared errors (MSE). Results are presented in Table 1. From Table 1, we observe that the maximum likelihood estimates for the QXg-F model with WBH-F are convergent.

**Table 1:** Bias and MSE of the MxLEs under the WBH-F.

n		Bias	MSE	Bias	MSE	Bias	MSE	Bias	MSE
		0%cens.		10%cens.		30%cens.		50%cens.	
20	$\rho$	0.94368	0.04958	0.927084	0.04857	0.895344	0.03958	0.9535934	0.04977
	K	0.83387	0.04758	0.800598	0.04932	0.818610	0.04109	0.764556	0.04067
	$\sigma ^{2}$	0.64890	0.04768	0.5782824	0.04674	0.5872129	0.02999	0.5856559	0.04908
	$\beta _{1}$	0.56165	0.04927	0.4936702	0.04919	0.4890512	0.04164	0.521098	0.04916
50	$\rho$	0.93728	0.04002	0.918837	0.04536	0.898274	0.03371	0.938401	0.04836
	K	0.83100	0.03995	0.800416	0.04784	0.81178	0.02511	0.788897	0.04004
	$\sigma ^{2}$	0.64201	0.04284	0.58813	0.04377	0.590121	0.02027	0.59604	0.04857
	$\beta _{1}$	0.55123	0.03879	0.495124	0.04509	0.496481	0.03366	0.520001	0.04806
300	$\rho$	0.92418	0.03648	0.910006	0.03486	0.899901	0.02546	0.917234	0.04799
	K	0.82431	0.02794	0.800372	0.02711	0.806742	0.02134	0.797896	0.04001
	$\sigma ^{2}$	0.62745	0.02451	0.591302	0.01937	0.595342	0.00996	0.598732	0.04777
	$\beta _{1}$	0.51935	0.01867	0.497301	0.02232	0.498004	0.01021	0.514638	0.04738
1000	$\rho$	0.91314	0.02374	0.900521	0.01658	0.90241	0.01340	0.905565	0.04500
	K	0.80658	0.02006	0.800142	0.01745	0.80186	0.02001	0.801247	0.03844
	$\sigma ^{2}$	0.61111	0.01865	0.61003	0.01008	0.602451	0.00618	0.599999	0.04627
	$\beta _{1}$	0.50041	0.01485	0.51008	0.00851	0.499989	0.00339	0.507430	0.03897

### Case of GBH-F

Using the GBH-F, the log-likelihood function for $\underline{ \textbf{P}}=(\kappa _{1},\rho _{1},\sigma ^{2},\beta )$ is given as follows:

$$\begin{aligned} \log L(\underline{\textbf{P}})= & {} r\log (\rho _{1})+\overset{n}{\underset{i=1 }{\sum }}\delta _{i}(\kappa _{1}t_{i}+x_{i}^{T}\underline{\varvec{\beta }})+ \overset{n}{\underset{i=1}{\sum }}\delta _{i}\log (3-2\sigma ^{2}+\sqrt{ {\mathbb {C}}\left[ \sigma ^{2}\right] }) \\{} & {} -\overset{n}{\underset{i=1}{\sum }}\delta _{i}\log (A_{2})+\sum \delta _{i}\log (B_{2})-\sum \delta _{i}\log \left( C_{2}\right) +\underset{i=1}{ \overset{n}{\sum }}\log (1+\sqrt{{\mathbb {C}}\left[ \sigma ^{2}\right] }) \\{} & {} +\underset{i=1}{\overset{n}{\sum }}\log (C_{2})-\underset{i=1}{\overset{n}{\sum }}\log (3-2\sigma ^{2}+\sqrt{{\mathbb {C}}\left[ \sigma ^{2}\right] })- \underset{i=1}{\overset{n}{\sum }}3\log (A_{2}). \end{aligned}$$

where

$$\begin{aligned} A_{2}= & {} \left( \frac{\rho _{1}}{\kappa _{1}}\right) (\exp (\kappa _{1}t_{i})-1)\exp (x_{i}^{T}\underline{\varvec{\beta }})(3-2\sigma ^{2}+ \sqrt{{\mathbb {C}}\left[ \sigma ^{2}\right] })+1+\sqrt{{\mathbb {C}}\left[ \sigma ^{2}\right] },\\ B_{2}= & {} \left[ \begin{array}{c} \left( 4-3\sigma ^{2}+\sqrt{{\mathbb {C}}\left[ \sigma ^{2}\right] }\right) \\ \times \left( \begin{array}{c} \left( \frac{\rho _{1}}{\kappa _{1}}\right) (\exp (\kappa _{1}t_{i})-1)\exp (x_{i}^{T}\underline{\varvec{\beta }})(3-2\sigma ^{2}+\sqrt{{\mathbb {C}}\left[ \sigma ^{2}\right] }) \\ +1+\sqrt{{\mathbb {C}}\left[ \sigma ^{2}\right] } \end{array} \right) ^{2} \end{array} \right] \\{} & {} +3\left( 1+\sqrt{{\mathbb {C}}\left[ \sigma ^{2}\right] }\right) ^{2}(\sigma ^{2}-1) \end{aligned}$$

and

$$\begin{aligned} C_{2}= & {} \left[ \begin{array}{c} \left( 4-3\sigma ^{2}+\sqrt{{\mathbb {C}}\left[ \sigma ^{2}\right] }\right) \\ \times \left( \begin{array}{c} \left( \frac{\rho _{1}}{\kappa _{1}}\right) (\exp (\kappa _{1}t_{i})-1)\exp (x_{i}^{T}\underline{\varvec{\beta }})(3-2\sigma ^{2} \\ +\sqrt{{\mathbb {C}}\left[ \sigma ^{2}\right] })+1+\sqrt{{\mathbb {C}}\left[ \sigma ^{2}\right] } \end{array} \right) ^{2} \end{array} \right] \\{} & {} +\left( 1+\sqrt{{\mathbb {C}}\left[ \sigma ^{2}\right] }\right) ^{2}(\sigma ^{2}-1). \end{aligned}$$

Maximizing the log-likelihood functions Eqs. (16) and (17), respectively, yields the appropriate MaxLE estimators $\widehat{\underline{\textbf{P}}}$ of parameter vectors $\underline{\textbf{P}}$. It is worth noting that $\widehat{\underline{\textbf{P}}}$ does not have a closed form. These optimization approaches are implemented in BBsolve R software packages (Ravi and Gilbert^31^).

### Simulations: case of GBH-F

We consider the QXg-F model with GBH-F. The data were simulated $N=13,000$ times; with parameter values$\kappa =1.07,$ $\rho =0.5,$ $\sigma ^{2}=0.7,$ $\beta _{1}=0.3$ and sample sizes $n=20,n=50,n=300$ and $n=1000$. Using the R software and the Barzilai-Borwein (BB) algorithm (Ravi and Gilbert^31^) for calculating the averages of the simulated values of the maximum likelihood estimators $\widehat{\kappa },\widehat{\rho },\widehat{\sigma ^{2}},\widehat{ \beta _{1}}$ parameters and their mean squared errors (MSE). Results are presented in Table 2. From Table 2, we observe that the maximum likelihood estimates for the QXg-F model with GBH-F are convergent.

**Table 2:** Bias and MSE of the MxLEs under the GBH-F.

n		Bias	MSE	Bias	MSE	Bias	MSE	Bias	MSE
		0% cens.		10% cens.		30% cens.		50% cens.	
20	$\rho$	0.52772	0.04752	0.4812452	0.03258	0.493951	0.03696	0.4934327	0.04985
	K	1.06838	0.02935	1.0691513	0.03564	1.0684768	0.04812	1.0695826	0.04721
	$\sigma ^{2}$	0.720627	0.01847	0.7326296	0.04446	0.7476699	0.02999	0.7235148	0.03954
	$\beta _{1}$	0.32221	0.04907	0.334851	0.04685	0.2834354	0.04770	0.2841911	0.04798
50	$\rho$	0.513308	0.03251	0.487938	0.01357	0.496684	0.03496	0.4971112	0.04405
	K	1.069354	0.01524	1.0693819	0.01985	1.0687684	0.03914	1.0697468	0.03216
	$\sigma ^{2}$	0.7162405	0.01847	0.717224	0.04021	0.7111120	0.01734	0.710743	0.01196
	$\beta _{1}$	0.317044	0.00965	0.328514	0.04612	0.293000	0.04382	0.290011	0.04380
300	$\rho$	0.508674	0.01358	0.489064	0.01003	0.4980094	0.02685	0.498946	0.03945
	K	1.072641	0.00758	1.0696649	0.01876	1.068888	0.01493	1.0698437	0.02999
	$\sigma ^{2}$	0.708657	0.00302	0.7063219	0.03681	0.706110	0.01112	0.6944002	0.01007
	$\beta _{1}$	0.309869	0.00063	0.312045	0.44378	0.298777	0.04128	0.290515	0.04268
1000	$\rho$	0.501102	0.00648	0.495876	0.00882	0.5120436	0.02506	0.499997	0.03945
	K	1.070043	0.00012	1.069994	0.00586	1.069889	0.01002	1.0699254	0.00177
	$\sigma ^{2}$	0.710030	0.00100	0.699589	0.02699	0.7003225	0.00659	0.700033	0.01001
	$\beta _{1}$	0.305201	0.00038	0.304061	0.44000	0.301066	0.04100	0.299999	0.04160

## Validating the QXg-F model using the NIK-RR test

There are many applications for the NIK-RR test statistic, making it a valuable tool for statistical analysis. It is particularly useful for selecting a model, assessing the goodness of fit of a model, and pinpointing problems with a model (more details may be found in Nikulin^13^, Nikulin^14^, Nikulin^15^, Nikulin^13^, and Rao and Robson^16^. The NIK-RR test statistic’s capacity to identify deviations from normalcy that other statistical tests might miss is one of its main features. Under the NIK-RR statistic, we need to test the following null hypothesis

$$\begin{aligned} H_{0}:\Pr \left\{ z_{i}\le z\right\} =F_{\underline{\textbf{P}}}(z),\hspace{ 0.2cm}z\in {\mathbb {R}} ,\hspace{0.2cm}\underline{\textbf{P}}=(\underline{\textbf{P}}_{1},\underline{ \textbf{P}}_{2},\cdots ,\underline{\textbf{P}}_{s})^{T}, \end{aligned}$$

Then, the NIK-RR statistic can be expressed as

$$\begin{aligned} Y^{2}(\widehat{\underline{\textbf{P}}}_{n})=X_{n}^{2}(\widehat{\underline{ \textbf{P}}}_{n})+\frac{1}{n}\varvec{\ell }^{T}(\widehat{\underline{\textbf{P }}}_{n})(\textbf{I}(\widehat{\underline{\textbf{P}}}_{n})-\textbf{J}( \widehat{\underline{\textbf{P}}}_{n}))^{-1}\varvec{\ell }(\widehat{ \underline{\textbf{P}}}_{n}), \end{aligned}$$

where

$$\begin{aligned} X_{n}^{2}(\underline{\textbf{P}})=\left( \left[ np_{1}(\underline{\textbf{P}} )\right] ^{-\frac{1}{2}}\left[ -np_{1}(\underline{\textbf{P}})+\underline{ \textbf{P}}_{1}\right] ,\cdots ,\left[ np_{b}(\underline{\textbf{P}})\right] ^{-\frac{1}{2}}\left[ -np_{b}(\underline{\textbf{P}})+\underline{\textbf{P}} _{b}\right] \right) ^{T} \end{aligned}$$

and

$$\begin{aligned} \textbf{J}(\underline{\textbf{P}})=B(\underline{\textbf{P}})^{T}B(\underline{ \textbf{P}}), \end{aligned}$$

refers to the information matrix where

$$\begin{aligned} {B(\underline{\textbf{P}}})=\left[ \frac{1}{\sqrt{p}_{i}}\frac{\partial }{ \partial \mu }(\underline{\textbf{P}})\right] _{r\times s}|i=1,2,\cdots ,b\ \text {and }\kappa =1,2,\cdots ,s, \end{aligned}$$

and

$$\begin{aligned} \varvec{\ell }(\underline{\textbf{P}})=(\varvec{\ell }_{1}(\underline{ \textbf{P}}),...,\varvec{\ell }_{s}(\underline{\textbf{P}}))^{T}\ \text {with} \hspace{0.2cm}\varvec{\ell }_{\kappa }(\underline{\textbf{P}})=\sum _{i=1}^{r} \frac{\underline{\textbf{P}}_{i}}{p_{i}}\frac{\partial p_{i}(\underline{ \textbf{P}})}{\partial \underline{\textbf{P}}_{\kappa }}, \end{aligned}$$

The $Y^{2}(\widehat{\underline{\textbf{P}}}_{n})$ statistic has $\left( b-1\right)$ degrees of freedom (DF) and is accompanied by the $\chi _{b-1}^{2}$ distribution, where the random sample. $x_{1},x_{2},\cdots ,x_{n}$ are collected in $\textbf{I}_{1},\textbf{I}_{2},\ldots ,\textbf{I}_{b}$ (these *b* subintervals are mutually disjoint: $\textbf{I}_{j}=]a_{j,b}-1;a_{j,b}]$ ). The intervals $\textbf{I}_{j}$’s limits for $a_{j,b}$ are determined as follows

$$\begin{aligned} p_{j}(\underline{\textbf{P}})=\int _{a_{j,b}-1}^{a_{j,b}}f_{\underline{ \textbf{P}}}\left( x\right) dx|\ j=1,2,\ldots ,b, \end{aligned}$$

and

$$\begin{aligned} a_{j,b}=F^{-1}\left( \frac{j}{b}\right) |j=1,\ldots ,b-1. \end{aligned}$$

### Uncensored assessing for the NIK-RR statistic

To verify the null hypothesis $H_{0}$ We thus produced the NIK-RR statistics of the QXg-F model to confirm that the sample is a 13000 using simulated samples n=25, n=50, n=150, n=350, n=550 and n=1000. Regarding various theoretical levels $\left( \epsilon =0.01,0.02,0.05,0.1\right)$, for the null hypothesis, we compute the average of the non-rejection numbers. $Y^{2}\le$ $\chi _{\epsilon }^{2}\left( b-1\right)$. The appropriate empirical and theoretical levels are presented in Table 3. It is evident that there is a good agreement between the calculated empirical level value and its equal theoretical level value. We therefore conclude that the proposed test is quite good for the QXg-F distribution.

**Table 3:** Uncensored assessing for the NIK-RR statistic for $\epsilon =0.01,0.02,0.05,0.1$ and $N=13000.$.

$n\downarrow \epsilon \longrightarrow$	$\epsilon =0.01$	$\epsilon =0.02$	$\epsilon =0.05$	$\epsilon =0.1$
$n=25$	0.99288	0.9825	0.9531	0.99286
$n=50$	0.99272	0.9820	0.9527	0.9021
$n=150$	0.9920	0.9813	0.9522	0.90117
$n=350$	0.9911	0.9809	0.95103	0.9008
$n=550$	0.9906	0.9805	0.9506	0.90044
$n=1000$	0.99032	0.98036	0.95054	0.9002

### Validation testing under the QXg-F and the B-NIK

According to Bagdonavicius and Nikulin^17^ and Bagdonavicius et al.^32^, one can verify the suitability of the QXg-F model when the parameters are unknown and the data are censored where null hypothesis can be expressed as

$$\begin{aligned} H_{0}:F(x)\in F_{0}=\left\{ F_{0}(x,\underline{\textbf{P}}),x\in R^{1},\ \underline{\textbf{P}}\in \underline{\textbf{P}}\subset R^{s}\right\} , \end{aligned}$$

Let’s divide the limited amount of time $[0,\tau ]$ into $\kappa |\kappa =1,2,\cdots ,s$ shorter time periods. Where is the maximum runtime of the research and $\textbf{I}_{j}=(a_{j-1},a_{j,b}];$ $0=<a_{0,b}<a_{1,b}...<a_{ \kappa -1,b}<a_{\kappa ,b}=+\infty .$ The anticipated worth of $\widehat{ a_{j,b}}$ can be said the following if $x_{(i)}$ is the $i^{th}$ element in the ordered statistics $(x_{(1)},,,x_{(n)})$ and if $\varvec{\Lambda }^{-1}$ refers to the cumulative hazard-rate function and

$$\begin{aligned} \widehat{a_{j,b}}=\varvec{\Lambda }^{-1}\left( (E_{j,X}-\sum _{l=1}^{i-1} \varvec{\Lambda }(x_{(l)},\widehat{\underline{\textbf{P}}}))/(n-i+1), \widehat{\underline{\textbf{P}}}\right) ,\ \ \ \widehat{a_{\kappa }} =x_{(n)}|_{\left( j=1,...,\kappa \right) }, \end{aligned}$$

where

$$\begin{aligned} e_{j,Z}= & {} E_{\kappa }/\kappa \text { for every }j.\\ \varvec{\Lambda }(x,\underline{\textbf{P}})= & {} -\ln \left[ \begin{array}{c} \left( 1+\sqrt{{\mathbb {C}}\left[ \sigma ^{2}\right] }\right) \\ \times \left[ \begin{array}{c} \left( 4-3\sigma ^{2}+\sqrt{{\mathbb {C}}\left[ \sigma ^{2}\right] }\right) \\ \times \left[ \left( \begin{array}{c} \Lambda _{0}(t_{i})\exp (x_{i}^{T}\underline{\varvec{\beta }}) \\ (3-2\sigma ^{2}+\sqrt{{\mathbb {C}}\left[ \sigma ^{2}\right] })+1+\sqrt{\mathbb { C}\left[ \sigma ^{2}\right] } \end{array} \right) ^{2}\right] \\ +\left( 1+\sqrt{{\mathbb {C}}\left[ \sigma ^{2}\right] }\right) ^{2}(\sigma ^{2}-1) \end{array} \right] \\ \times \left\{ (3-2\sigma ^{2}+\sqrt{{\mathbb {C}}\left[ \sigma ^{2}\right] } )\left( \begin{array}{c} \Lambda _{0}(t_{i})\exp (x_{i}^{T}\underline{\varvec{\beta }})(3-2\sigma ^{2}+\sqrt{{\mathbb {C}}\left[ \sigma ^{2}\right] }) \\ +1+\sqrt{{\mathbb {C}}\left[ \sigma ^{2}\right] } \end{array} \right) ^{3}\right\} ^{-1} \end{array} \right] , \end{aligned}$$

and

$$\begin{aligned} E_{j,Z}=(n-i+1)\varvec{\Lambda }(\widehat{a_{j,b}},\widehat{\underline{ \textbf{P}}})+\sum _{l=1}^{i-1}\varvec{\Lambda }(x_{(l)},\widehat{\underline{ \textbf{P}}})=\sum _{i:z_{i}>a_{j,b}}(\varvec{\Lambda }(a_{j,b}\wedge z_{i}, \widehat{\underline{\textbf{P}}})-\varvec{\Lambda }(a_{j-1},\widehat{ \underline{\textbf{P}}}),E_{\kappa }=\sum _{i=1}^{n}\varvec{\Lambda }(z_{i}, \widehat{\underline{\textbf{P}}}). \end{aligned}$$

The $a_{j,b}$ functions for random data, and the $e_{j,Z}$ For the $\kappa$ selected periods, anticipated failure rates are equal. Statistical data $Y_{n}^{2}=\textbf{Z}^{T}\widehat{\textbf{S}}^{-1}\textbf{Z}$, where $\textbf{Z}=(Z_{1},...,Z_{\kappa })^{x},$ $Z_{j}=\frac{1}{\sqrt{n}}(\textbf{W} _{j,Z}-e_{j,Z})|_{\left( \ j=1,2,...,\kappa \right) }$ and $\textbf{W}_{j,Z}$ can be used to test a hypothesis since it reflects the total number of failures that have been recorded throughout these time-shared. The elements of the B-NIK test statistic

$$\begin{aligned} Y_{n}^{2}=\sum _{j=1}^{\kappa }\frac{1}{\textbf{W}_{j,Z}}(\textbf{W} _{j,Z}-e_{j,Z})^{2}+\textbf{D}_{W,G} \end{aligned}$$

where

$$\begin{aligned} \textbf{D}_{W,G}= & {} \widehat{\textbf{V}}^{T}\widehat{\textbf{G}}^{-1} \widehat{\textbf{V}}, \\ \widehat{\textbf{S}}^{-1}= & {} \widehat{\textbf{B}}^{-1}+\widehat{\textbf{M}} ^{-1}\widehat{\textbf{B}}^{T}\widehat{\textbf{G}}^{-1}\widehat{\textbf{M}} \widehat{\textbf{B}}^{-1}, \\ \widehat{\textbf{G}}= & {} [\widehat{g}_{ll^{\prime }}]_{s\times s}=\widehat{i}- \widehat{\textbf{M}}\widehat{\textbf{B}}^{-1}\widehat{\textbf{M}}^{x}, \\ \widehat{\textbf{M}}_{lj}= & {} \frac{1}{n}\sum _{i:z_{i}\in \textbf{I}_{j}}\rho _{i}\frac{\partial }{\partial \underline{\textbf{P}}}\ln \left[ \lambda _{i, \underline{\textbf{P}}}(z_{i})\right] , \\ \textbf{W}_{j,Z}= & {} \sum _{i:z_{i}\in \textbf{I}_{j}}\rho _{i},\text { } \widehat{\textbf{B}}_{j}=n^{-1}\textbf{W}_{j,Z}, \\ \widehat{\textbf{V}}_{l}= & {} \sum _{j=1}^{\kappa }\widehat{\textbf{M}}_{lj} \widehat{\textbf{B}}_{j}^{-1}\textbf{Z}_{j},\ \ \ \ \ l,l^{\prime }=1,...,s,\\ \widehat{i}_{ll^{\prime }}= & {} n^{-1}\sum _{i=1}^{n}\rho _{i}\frac{ \partial }{\partial \underline{\textbf{P}}_{l}}\ln \left[ \lambda _{i, \underline{\textbf{P}}}(z_{i})\right] \frac{\partial }{\partial \underline{ \textbf{P}}_{l^{\prime }}}\ln \left[ \lambda _{i,\widehat{\underline{\textbf{ P}}}}(z_{i})\right] \end{aligned}$$

and

$$\begin{aligned} \widehat{g}_{ll^{\prime }}=\widehat{i}_{ll^{\prime }}-\sum _{j=1}^{\kappa } \widehat{\textbf{M}}_{lj}\widehat{\textbf{M}}_{l^{\prime }j}\hat{A}_{j}^{-1}, \end{aligned}$$

and

$$\begin{aligned} \widehat{\textbf{M}}_{lj}=\frac{1}{n}\sum _{i:z_{i}\in \textbf{I}_{j}}\rho _{i}\frac{\partial }{\partial \underline{\textbf{P}}}\ln \left[ \lambda _{i, \widehat{\underline{\textbf{P}}}}(z_{i})\right] . \end{aligned}$$

#### Censored-assessment under the B-NIK test

It is intended that the sample that was produced $\left( N=13000\right)$ will be censored at $25\%$ and that DF$=5$ To check if the sample agrees with the QXg-F model’s null hypothesis, grouping intervals will be used. For various theoretical levels, we determine the average value of the non-rejection numbers of the null hypothesis. $\left( \epsilon =0.01,0.02,0.05,0.1\right)$, where $Y^{2}\le$ $\chi _{\epsilon }^{2}\left( r-1\right)$. The theoretical and empirical levels are compared in Table 4, which demonstrates how closely the determined empirical level matches the value of the relevant theoretical level. We conclude that the customized test is ideally suited to the QXg-F model as a consequence.

**Table 4:** Censored assessing for the B-NIK statistic for $\epsilon =0.01;0.02;0.05;0.1$ and $N=13,000$.

$n\downarrow$ &$\epsilon \longrightarrow$	$\epsilon =0.01$	$\epsilon =0.02$	$\epsilon =0.05$	$\epsilon =0.1$
$n=25$	0.9925	0.9829	0.9532	0.9019
$n=50$	0.9920	0.9819	0.9525	0.9011
$n=150$	0.9914	0.9813	0.9519	0.9008
$n=350$	0.9909	0.9808	0.9510	0.9006
$n=550$	0.9905	0.9804	0.9505	0.9004
$n=1000$	0.9903	0.9803	0.9504	0.9002

We conclude from these findings that the empirical significance level of the $Y_{n}^{2}$ The theoretical significance level of the chi- square distribution with specific degrees of freedom aligns with the statistical significance level at which it becomes significant. Therefore, based on this evidence, the censored data obtained from the QXg-F distribution can be effectively fitted using the recommended test.

## The emergency care data

In medicine, frailty models are used to analyze the risk factors and prognosis of various diseases. They allow researchers to examine the impact of individual-level covariates (such as age, gender, and genetic factors) on patient outcomes, while also considering unmeasured or unobserved factors that may influence the risk of disease progression or mortality. Frailty models are often used in epidemiological studies, clinical trials, and studies involving patient cohorts to assess the effects of different treatments or interventions.

The emergency department of a the hospital of the public proximity health institution (Echatt, El tarf, Algeria), provided real data that were collected throughout the month of March 2023, which was used in the current study. The goal of this study was to examine, in a sample of people seeking medical care at the department, the relationship between various clinical variables and emergency room outcomes. The necessary approvals were obtained, and data collection was conducted under ethical guidelines. There were 30 different persons in the dataset, each of whom represented a distinct observation. Each person’s age (years), minimum and maximum blood pressure (mmHg), blood glucose level (mg/dL), cardiac frequency (BPM), and oxygen saturation (SaO2 %) were measured as six different variables. Strenuous measures were put in place during the data collection process to guarantee data quality and accuracy. This required accurate patient data documentation, adherence to established measurement techniques, and routine quality checks to catch any missing information or discrepancies. The thorough data collection process and the diversity of the patient population make this dataset valuable for examining the links between clinical factors and emergency room outcomes. By assessing the QXg-F model distribution’s goodness-of-fit and its capacity to faithfully represent the observed patterns and variability in emergency care data, we are able to explore the validity and application of the distribution. We present the point estimates for each fitted model (QXg-F model with Weibull baseline hazard-rate function and QXg-F model with Gompertz baseline hazard-rate function). The well known modified chi-squared test (see^17^) is supplied to identify the best model among all fitted models to this data.

### Validation of the QXg-F model under the Weibull baseline hazard-rate function

Assuming that these data are distributed according to the QXg-F model with Weibull baseline hazard-rate function. Then, using R statistical software (the BB package), the maximum likelihood estimates of the parameter vector $\underline{\textbf{P}}$ are obtained as

$$\begin{aligned} \widehat{\kappa _{1}}= & {} (0.93359597,\widehat{\rho _{1}}=0.92339747,\widehat{ \sigma ^{2}}=1.05033488, \\ \widehat{\beta _{1}}= & {} 0.09416582,\widehat{\beta _{2}}=0.12605622,\widehat{ \beta _{3}}=-0.10260262, \\ \beta _{4}= & {} -0.43375125,\widehat{\beta _{5}}=0.21897114,\widehat{\beta _{6} }=0.46708022). \end{aligned}$$

According to Bagdonavičius and Nikulin^17^ for censored data, we take for example 5 intervals $\left( r=5\right)$ as number of classes. The elements of the estimated Fisher information matrix $I\left( \widehat{ \underline{\textbf{P}}}\right)$ are presented as follows:

$$\begin{aligned} I\left( \widehat{\underline{\textbf{P}}}\right) =\left( \begin{array}{ccccccccc} 1.10224 &{} -2.85216 &{} 0.35487 &{} -3.2015 &{} 5.12410 &{} -2.6585 &{} 0.75482 &{} -3.0002 &{} 1.95243 \\ &{} 0.982451 &{} -6.3214 &{} 2.73618 &{} 0.36485 &{} 0.51247 &{} -1.6832 &{} -4.12574 &{} 1.55505 \\ &{} &{} 0.62541 &{} 2.93485 &{} -6.3298 &{} 0.66485 &{} -21.5348 &{} 2.87361 &{} -7.0164 \\ &{} &{} &{} 1.32048 &{} 0.00843 &{} 1.31405 &{} 0.90340 &{} 1.11619 &{} -9.3761 \\ &{} &{} &{} &{} 2.96511 &{} -4.0527 &{} 1.6233 &{} 0.61372 &{} 0.8391 \\ &{} &{} &{} &{} &{} 0.84755 &{} 2.0006 &{} 1.86254 &{} 0.37770 \\ &{} &{} &{} &{} &{} &{} 0.32651 &{} -8.58102 &{} 1.7438 \\ &{} &{} &{} &{} &{} &{} &{} 1.70015 &{} -10.3254 \\ &{} &{} &{} &{} &{} &{} &{} &{} 0.74006 \end{array} \right) \end{aligned}$$

Then we calculate the value of the test statistic as $Y_{n}^{2}=8.076495.$ The critical value is $\chi _{0.05}^{2}(4)=9.488>Y_{n}^{2},.$ This data can be fitted by our proposed QXg-F model with Weibull baseline hazard-rate function in proper manner.

### Validation of the QXg-F model under the Gompertz baseline hazard-rate function

Assuming that these data are distributed according to the QXg-F model with Gompertz baseline hazard-rate function. Then, using R statistical software (the BB package), the maximum likelihood estimator of the parameter vector $\underline{\textbf{P}}$ can be obtained as

$$\begin{aligned} \widehat{\kappa }= & {} 1.00593154,\widehat{\rho }=0.74417604,\widehat{\sigma ^{2}}=1.07010909, \\ \widehat{\beta _{1}}= & {} 0.16228331,\widehat{\beta _{2}}=0.17405692,\widehat{ \beta _{3}}=0.02678224, \\ \widehat{\beta _{4}}= & {} -0.56805721,\widehat{\beta _{5}}=-0.07338661, \widehat{\beta _{6}}=0.44793647. \end{aligned}$$

We take $r=5$ intervals and the estimated Fisher matrix expressed as

$$\begin{aligned} I\left( \widehat{\underline{\textbf{P}}}\right) =\left( \begin{array}{ccccccccc} 2.6352 &{} -3.6257 &{} 1.0654 &{} 0.8475 &{} -6.3258 &{} -3.1547 &{} 2.0001 &{} 0.9321 &{} 1.8547 \\ &{} 0.9658 &{} 0.8475 &{} 1.8654 &{} 1.3274 &{} -3.9547 &{} -8.1755 &{} -11.132 &{} 1.9584 \\ &{} &{} 1.3475 &{} 2.0084 &{} 0.6254 &{} 1.3648 &{} -2.3647 &{} 0.6845 &{} 0.9614 \\ &{} &{} &{} 0.6321 &{} -9.3020 &{} 3.0001 &{} 1.0854 &{} 0.6845 &{} -3.628 \\ &{} &{} &{} &{} 1.6847 &{} 1.0054 &{} 0.3754 &{} 1.0024 &{} 1.9045 \\ &{} &{} &{} &{} &{} 0.3617 &{} 1.6584 &{} 0.7845 &{} 1.0325 \\ &{} &{} &{} &{} &{} &{} 0.8647 &{} 0.1254 &{} 0.9658 \\ &{} &{} &{} &{} &{} &{} &{} 1.0954 &{} 1.8457 \\ &{} &{} &{} &{} &{} &{} &{} &{} 1.3643 \end{array} \right) , \end{aligned}$$

then we calculate the value of the Bagdonavičius and Nikulin^17^ statistic : $Y_{n}^{2}=7.43821106$. For different critical values : $\alpha =5\%$ and $\alpha =10\%$, we find $Y^{2}<\chi _{0.05}^{2}\left( 5-1\right) =9.488$ and $Y^{2}<\chi _{0.1}^{2}\left( 5-1\right) =7.779$ respectively. Hence we reason that the emergency care data is compatible with our proposed QXg-F model with Gompertz baseline hazard-rate function.

## Testing the ability of the new QXg-F model in risk analysis

In risk analysis, frailty models can be used to predict the probability of an event taking place, such as the risk of developing a specific disease or suffering a specific adverse event, while also taking into consideration individual characteristics that may influence how the risk is affected. To model risks and to construct models for risk prediction, fragility models have been utilized in a wide variety of sectors, including epidemiology, medical research, and actuarial science, amongst others. The use of frailty models can provide more accurate estimates of risk and increase understanding of the underlying mechanisms that determine the risk of an occurrence. Both of these benefits can be realized simultaneously. In the fields of insurance and actuarial science, frailty models are applied in order to take into account the fact that individuals’ rates of death are very variable. This variation may be attributable to a wide range of factors, such as age, gender, current health state, lifestyle choices, and genetic predisposition. Insurers can more properly price their insurance products using frailty models, and they can also better control the risks to which they are exposed. In the fields of insurance and actuarial science, some of the specific uses of frailty models include the following:

1. It is possible to employ frailty models in order to estimate the probable future mortality of a group of individuals, which may subsequently be used in order to price life insurance premiums.
2. The present value of an annuity is the amount of money that an individual would need to invest today in order to obtain a guaranteed income stream for the rest of their life. Frailty models can be used to estimate the present value of an annuity. This is the amount of money that an individual would need to invest today in order to acquire this income stream.
3. Identifying and managing the risks that are connected to a specific insurance product or portfolio of products can be accomplished with the assistance of frailty models. To identify high-risk individuals who are more prone to file claims, an insurer might, for instance, utilize a frailty model.
4. The application of fragility models is a powerful instrument that has the potential to enhance the precision and effectiveness of insurance pricing as well as risk management. Frailty models are anticipated to become increasingly more commonly employed in the insurance sector as the discipline of actuarial science continues to develop and progress.

The application of frailty models in the fields of actuarial science and insurance provides the following additional benefits:

1. Insurers may benefit from using frailty models since it helps them gain a better understanding of the risk of loss connected with a certain individual or group of individuals. This can lead to decisions regarding pricing and underwriting that are more accurate.
2. Insurance companies can build solutions that are more suited to meet the requirements of individual clients with the use of frailty models. This may result in improved levels of satisfaction and loyalty among customers.
3. Insurers can automate a significant portion of the work that is related with risk assessment and pricing with the use of frailty models. This may result in cost savings as well as enhanced operational efficacy.

### Risk indicators for the new QXg-F model

It is not necessary to supply any further characterization of risk exposure beyond what may be provided by probability-based distributions. Most of the time, one value or at the very least a small collection of numbers is used to describe the level of risk exposure. These data on risk exposure are plainly functions of a particular model. They are commonly referred to as important key risk indicators (KRIs), which is an abbreviation for key risk indicator. Actuaries and risk managers commonly concentrate their efforts on evaluating the possibility of an adverse outcome, which can be communicated through the use of the V-R indicator at a specific probability or confidence level.

This indicator is frequently used to calculate the amount of capital needed to deal with such probable negative situations. The V-R of the QXg-F model at the $100q\%$ level, say V-R$\left( Z;\underline{\textbf{P}}\right)$ or $\pi \left( q\right)$, is the $100q\%$ quantile (or percentile). Then, we can simply write

16 $$\begin{aligned} \text {V-R}\left( Z;\underline{\textbf{P}}\right) =\Pr \left( Z>Q\left( U\right) \right) = {\left\{ \begin{array}{ll} 1\%|_{q=99\%} \\ 5\%|_{q=95\%} \\ \vdots \end{array}\right. }, \end{aligned}$$

where $Q\left( U\right)$ is quantile function of the QXg-F model, for a one-year time when $q=99\%$, the interpretation is that there is only a very small chance ($1\%$) that the insurance company will be bankrupted by an adverse outcome over the next year. Generally speaking, if the distribution of gains (or losses) is limited to the normal distribution, it is acknowledged that the number V-R$\left( Z;\underline{\textbf{P}}\right)$ meets all coherence requirements. The data sets for insurance such as the insurance claims and reinsurance revenues are typically skewed whether to the right or to the left , though. Using the normal distribution to describe the revenues from reinsurance and insurance claims is not suitable. The TLV-R $\left( Z;\underline{\textbf{P}}\right)$ of *Z* at the $100q\%$ confidence level is the expected loss given that the loss exceeds the $100q\%$ of the distribution of *Z*, then the TLV-R of *Z* can be expressed as

17 $$\begin{aligned} \text {TLV-R}\left( Z;\underline{\textbf{P}}\right) ={{\mathbb {E}}} \left( X|X>\pi \left( q\right) \right) =\frac{1}{1-F_{\underline{\textbf{V}} }\left( \pi \left( q\right) \right) }\int \limits _{\pi \left( q\right) }^{\infty }z\,f_{\underline{\textbf{P}}}\left( z\right) dz=\frac{1}{1-q} \int \limits _{\pi \left( q\right) }^{\infty }z\,f_{\underline{\textbf{P}} }\left( z\right) dz. \end{aligned}$$

The quantity TLV-R$\left( Z;\underline{\textbf{P}}\right)$, which gives further details about the tail of the QXg-F distribution, is therefore the average of all the V-R values mentioned above at the confidence level *q*. Moreover, the TLV-R(*Z*) can also be expressed as TLV-R$\left( Z;\underline{ \textbf{P}}\right) =e\left( Z;\underline{\textbf{P}}\right) +$V-R$\left( Z; \underline{\textbf{P}}\right) ,$ where $e\left( Z;\underline{\textbf{P}} \right)$ is the mean excess loss (M-EXL$\left( Z;\underline{\textbf{P}} \right)$) function evaluated at the $100q\%^{th}$ quantile (see^33–35^). When the $e\left( Z;\underline{ \textbf{P}}\right)$ value vanishes, then TLV-R$\left( Z;\underline{\textbf{P }}\right) =$V-R$\left( Z;\underline{\textbf{P}}\right)$ and for the very small values of $e\left( Z;\underline{\textbf{P}}\right)$, the value of TLV-R$\left( Z;\underline{\textbf{P}}\right)$ will be very close to V-R$\left( Z;\underline{\textbf{P}}\right) .$The T-VC risk indicator, which Furman and Landsman^36^ developed, calculates the loss’s deviation from the average along a tail. Explicit expressions for the T-VC risk indicator under the multivariate normal distribution were also developed by Furman and Landsman^36^. The T-VC risk indicator (T-VC$\left( Z;\underline{\textbf{P} }\right)$) can then be expressed as

18 $$\begin{aligned} \text {T-VC}\left( Z;\underline{\textbf{P}}\right) = {{\mathbb {E}}}\left( Z^{2}|Z>\pi \left( q\right) \right) -\left[ \text {TLV-R}(Z)\right] ^{2}. \end{aligned}$$

As a statistic for the best portfolio choice, Landsman^37^ developed the TMVK risk indicator, which is based on the T-VC risk indicator. Consequently, the TMVK risk indicator may be written as

19 $$\begin{aligned} \text {TMVK}(X)=\text {TLV-R}(Z)+\pi \text {T-VC}(Z)|_{0<\pi <1}. \end{aligned}$$

Then, for any RV, TMVK$\left( Z;\underline{\textbf{P}}\right)>$T-VC$\left( Z;\underline{\textbf{P}}\right)$ and, for $\pi =1$, TMVK$\left( Z; \underline{\textbf{P}}\right) =$TLV-R$\left( Z;\underline{\textbf{P}}\right) .$

### Assessing under different estimation methods via simulations

In this section, we assess the MaxLE, the OLS, the WLSE, the AnDE methods for calculating the KRIs. These quantities are estimated using $N=1,000$ with different sample sizes $(n=20,50,100)$ and three confidence levels (C-Ls) ($q=(50\%,60\%,70\%,90\%,99\%)$). All results are reported in Table 5 (KRIs under artificial data for n=20), Table 6 (KRIs under artificial data for n=50) and Table 7 (KRIs under artificial data for n=100) from which we conclude: V-R$\left( Z;\underline{\textbf{P}}\right)$, TLV-R$\left( Z; \underline{\textbf{P}}\right)$ and TMVK$\left( Z;\underline{\textbf{P}} \right)$ increase when *q* increases for all estimation methods. V-R$\left( Z;\underline{\textbf{P}}\right) _{\text {WLS}}<$V-R$\left( Z;\underline{ \textbf{P}}\right) _{\text {AnDE}}<$V-R$\left( Z;\underline{\textbf{P}} \right) _{\text {MaxLE}}<$ V-R$\left( Z;\underline{\textbf{P}}\right) _{\text { OrLSE}}$ for most *q*. TLV-R$\left( Z;\underline{\textbf{P}}\right) _{\text { WLS}}<$TLV-R$\left( Z;\underline{\textbf{P}}\right) _{\text {AnDE}}<$TLV-R$\left( Z;\underline{\textbf{P}}\right) _{\text {MaxLE}}<$TLV-R$\left( Z; \underline{\textbf{P}}\right) _{\text {OrLSE}}$ for most *q*.

**Table 5:** KRIs under artificial data for n=20.

Method	$\widehat{\zeta }$	$\widehat{\theta }$	V-R$\left( Z;\underline{ \textbf{P}}\right)$	TLV-R$\left( Z;\underline{\textbf{P}}\right)$	T-VC$\left( Z;\underline{\textbf{P}}\right)$	TMVK$\left( Z;\underline{\textbf{P }}\right)$	MELS$\left( Z;\underline{\textbf{P}}\right)$
MaxLE	0.14107,0.52017						
50%			4.6982252	7.9207537	8.1291089	11.9853081	3.2225285
60%			5.5534017	8.6216855	7.6896604	12.4665157	3.0682838
70%			6.5570515	9.4827166	7.2595049	13.112469	2.9256651
80%			7.8541408	10.6385741	6.8116389	14.0443935	2.7844333
90%			9.8829563	12.5095433	6.2864654	15.652776	2.626587
95%			11.769106	14.2920432	5.9283057	17.256196	2.5229372
99%			15.8459571	18.2225931	5.4063186	20.9257524	2.376636
OrLSE	0.25768,0.49028						
50%			4.6424595	8.1062567	9.3000559	12.7562847	3.4637972
60%			5.5685106	8.8587309	8.7761598	13.2468108	3.2902204
70%			6.6502786	9.7810246	8.2666565	13.9143528	3.1307459
80%			8.0427591	11.0166329	7.7395836	14.8864248	2.9738738
90%			10.2129492	13.0127723	7.1258076	16.5756761	2.7998232
95%			12.2254069	14.911675	6.7097639	18.2665569	2.6862681
99%			16.5664285	19.0933974	6.1069945	22.1468946	2.5269688
WLSE	0.27324,0.48524						
50%			4.6474756	8.1526927	9.5131474	12.9092664	3.5052172
60%			5.5853582	8.9140626	8.9747332	13.4014292	3.3287043
70%			6.6804225	9.8470259	8.4514401	14.072746	3.1666034
80%			8.0893887	11.096634	7.9104722	15.0518701	3.0072453
90%			10.2843424	13.1149134	7.2810078	16.7554173	2.830571
95%			12.3191384	15.0345255	6.8546307	18.4618409	2.7153871
99%			16.7072472	19.2611659	6.2373245	22.3798281	2.5539186
CVM	0.24569,0.49695						
50%			4.6131432	8.0262669	9.0380238	12.5452788	3.4131237
60%			5.5250504	8.7678136	8.5307887	13.033208	3.2427632
70%			6.5907158	9.676896	8.0372303	13.6955112	3.0861803
80%			7.9629605	10.8950332	7.526371	14.6582187	2.9320728
90%			10.102331	12.8633142	6.9310958	16.3288621	2.7609832
95%			12.0866897	14.7359852	6.5273691	17.9996698	2.6492955
99%			16.3679401	18.8604668	5.9421197	21.8315266	2.4925267

**Table 6:** KRIs under artificial data for n=50.

Method	$\widehat{\zeta }$	$\widehat{\theta }$	V-R$\left( Z;\underline{ \textbf{P}}\right)$	TLV-R$\left( Z;\underline{\textbf{P}}\right)$	T-VC$\left( Z;\underline{\textbf{P}}\right)$	TMVK$\left( Z;\underline{\textbf{P }}\right)$	MELS$\left( Z;\underline{\textbf{P}}\right)$
MaxLE	0.18714,0.50706						
50%			4.6845211	8.0084233	8.6125268	12.3146867	3.3239022
60%			5.5694373	8.7310154	8.1386873	12.800359	3.161578
70%			6.6058505	9.6178117	7.6762239	13.4559236	3.0119612
80%			7.9429961	10.8072504	7.1960754	14.4052881	2.8642543
90%			10.0313044	12.7310065	6.6347243	16.0483686	2.699702
95%			11.9707151	14.5626636	6.2528722	17.6890997	2.5919485
99%			16.1591777	18.5994275	5.6977361	21.4482955	2.4402497
OrLSE	0.21376,0.49717						
50%			4.7007731	8.1008547	8.9916608	12.5966851	3.4000817
60%			5.6075032	8.8397982	8.492301	13.0859487	3.2322951
70%			6.668335	9.7462022	8.0056485	13.7490265	3.0778672
80%			8.0357406	10.9613828	7.5011306	14.711948	2.9256421
90%			10.1695559	12.9258941	6.9122092	16.3819986	2.7563381
95%			12.1500806	14.7957162	6.5121632	18.0517978	2.6456356
99%			16.4253531	18.9153562	5.9313583	21.8810353	2.4900031
WLSE	0.22011,0.49643						
50%			4.689736	8.0972504	9.0263673	12.6104341	3.4075144
60%			5.5987983	8.8377616	8.5239999	13.0997615	3.2389633
70%			6.662104	9.7459829	8.034577	13.7632714	3.083879
80%			8.0324131	10.9634708	7.5273556	14.7271486	2.9310577
90%			10.1703532	12.9315076	6.9354919	16.3992535	2.7611544
95%			12.1544387	14.8045359	6.5335763	18.071324	2.6500971
99%			16.4369338	18.9309503	5.9502389	21.9060698	2.4940165
CVM	0.20859,0.49996						
50%			4.6892727	8.0684722	8.8853283	12.5111364	3.3791995
60%			5.5901462	8.8029166	8.3927509	12.999292	3.2127704
70%			6.6443362	9.7038884	7.9125764	13.6601766	3.0595522
80%			8.0034148	10.9118918	7.41464	14.6192118	2.908477
90%			10.1245679	12.8649676	6.8332241	16.2815797	2.7403997
95%			12.0935586	14.7240273	6.4381719	17.9431133	2.6304687
99%			16.3443116	18.8201913	5.8644697	21.7524262	2.4758797

**Table 7:** KRIs under artificial data for n=100.

Method	$\widehat{\zeta }$	$\widehat{\theta }$	V-R$\left( Z;\underline{ \textbf{P}}\right)$	TLV-R$\left( Z;\underline{\textbf{P}}\right)$	T-VC$\left( Z;\underline{\textbf{P}}\right)$	TMVK$\left( Z;\underline{\textbf{P }}\right)$	MELS$\left( Z;\underline{\textbf{P}}\right)$
MaxLE	0.1943,0.50444						
50%			4.6882397	8.0320981	8.7108906	12.3875434	3.3438585
60%			5.5788848	8.7589716	8.2304017	12.8741725	3.1800868
70%			6.6216992	9.650898	7.7616432	13.5317196	3.0291987
80%			7.9667667	10.8470672	7.2751553	14.4846448	2.8803005
90%			10.0669753	12.7814728	6.7066399	16.1347928	2.7144975
95%			12.0171294	14.6230983	6.3200621	17.7831293	2.6059689
99%			16.2282628	18.6815004	5.7582622	21.5606315	2.4532376
OrLSE	0.21376,0.49885						
50%			4.6849052	8.0735006	8.9310096	12.5390054	3.3885954
60%			5.5885719	8.8099477	8.4350185	13.027457	3.2213759
70%			6.6458198	9.7132897	7.9516488	13.6891141	3.0674699
80%			8.008606	10.924365	7.4505375	14.6496338	2.915759
90%			10.1352129	12.8822401	6.8655888	16.3150345	2.7470271
95%			12.1090473	14.7457459	6.4682413	17.9798666	2.6366986
99%			16.3698779	18.8514699	5.8913542	21.797147	2.481592
WLSE	0.21105,0.49985						
50%			4.6833079	8.0641711	8.8922752	12.5103087	3.3808631
60%			5.5847622	8.7989583	8.3988943	12.9984054	3.2141961
70%			6.6395315	9.7003094	7.9179995	13.6593092	3.0607779
80%			7.9992451	10.9087709	7.419382	14.6184619	2.9095258
90%			10.1212311	12.8625078	6.8372537	16.2811347	2.7412767
95%			12.0908908	14.7221388	6.4417673	17.9430224	2.6312479
99%			16.3429079	18.8194491	5.8675051	21.7532016	2.4765411
CVM	0.21109,0.50028						
50%			4.6791079	8.0570562	8.8769135	12.495513	3.3779483
60%			5.5797877	8.7912095	8.3843767	12.9833979	3.2114219
70%			6.6336488	9.6917823	7.904306	13.6439353	3.0581335
80%			7.9921894	10.8991994	7.4065397	14.6024693	2.90701
90%			10.1123418	12.8512459	6.8254163	16.263954	2.738904
95%			12.0802975	14.7092668	6.4306107	17.9245721	2.6289693
99%			16.3286324	18.8030275	5.8573383	21.7316967	2.4743951

### Assessing under different estimation methods via insurance data

As a concrete example, in this section of the essay, we take a look at the insurance claims payment triangle from the perspective of a United Kingdom Motor Non-Comprehensive account. We find the years 2007 to 2013 to be the most convenient origin era. The data on the claims are presented within the insurance claims payment data frame in the format that one would typically find it organised within a database. The first column contains information pertaining to the origin year, which can fall anywhere between 2007 and 2013, the development year, as well as the incremental payments. It is essential to point out that the data on insurance claims were initially analysed using a probability-based distribution. This was done in the beginning of the process. The ability of the insurance company to deal with events of this nature is something that actuaries, regulators, investors, and rating agencies all place a high level of weight on. This study suggests a number of KRI quantities for the left-skewed insurance claims data based on the QXg-F distribution. These quantities include VAR, TVAR, T-VC, and TM-V (see^38^ for more information). In Table 8, the KRIs that are included under the insurance calculations data are broken down according to each and every estimating method used for the QXg-F model.

Based on Table 8, the following results can be highlighted:

1. In general, whatever the risk assessment method:
  $$\begin{aligned} \text {V-R}(Z;\widehat{\underline{\textbf{P}}}|1-q= & {} 50\%)<\text {V-R}(Z; \widehat{\underline{\textbf{P}}}|1-q=40\%) \\< & {} ...<\text {V-R}(Z;\widehat{\underline{\textbf{P}}}|1-q=5\%)<\text {V-R}(Z; \widehat{\underline{\textbf{P}}}|1-q=1\%). \end{aligned}$$
2. Also,
  $$\begin{aligned} \text {TLV-R}(Z;\widehat{\underline{\textbf{P}}}|1-q= & {} 50\%)<\text {TLV-R}(Z; \widehat{\underline{\textbf{P}}}|1-q=40\%) \\< & {} ...<\text {TLV-R}(Z;\widehat{\underline{\textbf{P}}}|1-q=5\%)<\text {TLV-R} (Z;\widehat{\underline{\textbf{P}}}|1-q=1\%). \end{aligned}$$
3. In general, whatever the risk assessment method:
  $$\begin{aligned} \text {T-VC}(Z;\widehat{\underline{\textbf{P}}}|1-q= & {} 50\%)>\text {T-VC}(Z; \widehat{\underline{\textbf{P}}}|1-q=40\%) \\> & {} ...>\text {T-VC}(Z;\widehat{\underline{\textbf{P}}}|1-q=5\%)>\text {T-VC}(Z; \widehat{\underline{\textbf{P}}}|1-q=1\%). \end{aligned}$$
4. Moreover
  $$\begin{aligned} \text {TMVK}(Z;\widehat{\underline{\textbf{P}}}|1-q= & {} 50\%)>\text {TMVK}(Z; \widehat{\underline{\textbf{P}}}|1-q=40\%) \\> & {} ...>\text {TMVK}(Z;\widehat{\underline{\textbf{P}}}|1-q=5\%)>\text {TMVK}(Z; \widehat{\underline{\textbf{P}}}|1-q=1\%). \end{aligned}$$
5. Finally
  $$\begin{aligned} \text {MELS}(Z;\widehat{\underline{\textbf{P}}}|1-q= & {} 50\%)>\text {MELS}(Z; \widehat{\underline{\textbf{P}}}|1-q=40\%) \\> & {} ...>\text {MELS}(Z;\widehat{\underline{\textbf{P}}}|1-q=5\%)>\text {MELS}(Z; \widehat{\underline{\textbf{P}}}|1-q=1\%). \end{aligned}$$
6. Under the MaxLE method: The V-R$\left( Z;\widehat{\underline{\textbf{P} }}\right)$ is monotonically increasing indicator, the TLV-R$\left( Z; \widehat{\underline{\textbf{P}}}\right)$ is increases steadily and continuously. However the T-VC$\left( Z;\widehat{\underline{\textbf{P}}}\right)$, the TMVK$\left( Z;\widehat{\underline{\textbf{P}}}\right)$ and the MELS$\left( Z;\widehat{\underline{\textbf{P}}}\right)$ are increases steadily and continuously.
7. Under the OrLSE method: The V-R$\left( Z;\widehat{\underline{\textbf{P} }}\right)$ is monotonically increasing indicator, the TLV-R$\left( Z; \widehat{\underline{\textbf{P}}}\right)$ is increases steadily and continuously. However the T-VC$\left( Z;\widehat{\underline{\textbf{P}}}\right)$, the TMVK$\left( Z;\widehat{\underline{\textbf{P}}}\right)$ and the MELS$\left( Z;\widehat{\underline{\textbf{P}}}\right)$ are increases steadily and continuously.

**Table 8:** KRIs under artificial data.

Method	$\widehat{\zeta }$	$\widehat{\theta }$	V-R$\left( Z;\underline{ \textbf{P}}\right)$	TLV-R$\left( Z;\underline{\textbf{P}}\right)$	T-VC$\left( Z;\underline{\textbf{P}}\right)$	TMVK$\left( Z;\underline{\textbf{P }}\right)$	MELS$\left( Z;\underline{\textbf{P}}\right)$
MaxLE	0.056648,0.00107						
50%			2408.285176	3957.286678	1894484.692667	951199.633011	1549.001501
60%			2816.579287	4294.591947	1795766.569883	902177.876889	1478.012661
70%			3297.89361	4709.755039	1698501.924311	853960.717195	1411.861429
80%			3922.142321	5268.031519	1596614.004881	803575.03396	1345.889198
90%			4901.547418	6173.031474	1478243.31119	745294.687069	1271.484056
95%			5814.002477	7036.477935	1395540.819773	704806.887822	1222.475459
99%			7789.512693	8942.216564	1278264.671458	648074.552293	1152.703872
OrLSE	0.272949,0.000899						
50%			2509.021497	4400.995807	2771619.894328	1390210.942971	1891.97431
60%			3015.24518	4811.953608	2614766.380651	1312195.143934	1796.708428
70%			3606.312937	5315.532936	2462318.334862	1236474.700367	1709.219999
80%			4366.817414	5990.028363	2304719.229173	1158349.64295	1623.210949
90%			5551.576191	7079.430874	2121334.380204	1067746.620976	1527.854683
95%			6649.89369	8115.579457	1997119.012415	1006675.085665	1465.685767
99%			9018.464769	10396.999002	1817273.115655	919033.556829	1378.534233
WLSE	0.13893,0.001019						
50%			2402.375177	4047.542671	2119132.335979	1063613.710661	1645.167494
60%			2838.891506	4405.392352	2004673.486117	1006742.13541	1566.500846
70%			3351.247105	4844.998236	1892621.739739	951155.868105	1493.751131
80%			4013.456607	5435.154825	1775934.490963	893402.400306	1421.698218
90%			5049.313766	6390.469523	1639088.326576	825934.632811	1341.155757
95%			6012.378498	7300.638908	1545748.650037	780174.963926	1288.26041
99%			8094.095078	9307.560124	1412012.076223	715313.598236	1213.465046
CVM	0.215302,0.000933						
50%			2503.029113	4315.472068	2554671.581296	1281651.262716	1812.442955
60%			2986.414861	4709.366036	2412721.162258	1211069.947165	1722.951174
70%			3551.920461	5192.511163	2274394.097108	1142389.559717	1640.590702
80%			4280.816611	5840.228281	2130999.793612	1071340.125087	1559.411671
90%			5418.195151	6887.328172	1963634.040249	988704.348297	1469.133022
95%			6473.831185	7883.938659	1849952.187187	932860.032253	1410.107475
99%			8752.526684	10079.658969	1684916.244941	852537.78144	1327.132285

## Discussion

In this study, we proposed a novel quasi xgamma frailty (QXg-F) model for survival analysis. The QXg-F model aims to capture the complex interplay between frailty and survival outcomes, providing a more comprehensive understanding of the heterogeneity present in the data. To assess the suitability of the QXg-F model, we employ the Nikulin-Rao-Robson goodness-of-fit test, which evaluates how well the model’s distribution fits the observed data.

Our investigation extends beyond model development to include an in-depth examination of the QXg-F model’s properties and performance compared to other commonly used distributions in frailty modeling. Through simulation studies and real data applications, including a novel dataset from an emergency hospital in Algeria, we demonstrate the QXg-F model’s ability to accurately capture heterogeneity and improve model fit. Our findings suggest that the QXg-F model represents a promising alternative to existing frailty modeling distributions, with potential applications in various sectors, including emergency care.

Furthermore, we explore the utility and significance of the QXg-F model in insurance settings. By conducting simulations and applying the model to insurance data, we assess its performance and relevance in predicting survival outcomes and estimating risk in insurance contexts. This analysis provides valuable insights into the QXg-F model’s applicability beyond healthcare, highlighting its potential to enhance risk assessment and decision-making in insurance-related scenarios. Here are a few potential limitations that could be addressed:

- The study may rely on certain assumptions about the underlying data distribution or the relationship between variables. It would be beneficial to validate these assumptions rigorously, as any inaccuracies or deviations could impact the validity of the findings.
- The study’s conclusions may be based on a specific dataset, such as emergency care data, which could limit the generalizability of the results. Assessing the robustness of the findings across different datasets or populations would enhance the study’s applicability and relevance.
- Introducing a novel model like the QXg-F model may add complexity to the analysis. While sophisticated models can offer valuable insights, they may also be more challenging to interpret and implement in practice. It would be helpful to provide clear explanations and guidelines for applying the model in real-world scenarios.
- If the study includes risk analysis for emergency care data, it is essential to transparently outline the assumptions and methodologies used in the risk assessment. This ensures that stakeholders can critically evaluate the risk estimates and understand the associated uncertainties.
- The analysis may overlook external factors or confounding variables that could influence the survival outcomes or introduce bias into the results. Accounting for potential confounders and conducting sensitivity analyses would enhance the study’s validity and reliability.

## Conclusions and future points

This work suggested and analysed a flexible frailty model called as the quasi-Xgamma frailty (QXg-F) probapility model for statistical modeling of the unobserved-heterogeneity in survival and reliability data sets. We established both unconditional survival and hazard functions when we calculated the Laplace transform of this frailty distribution. The baseline hazard functions used to construct the two QXg-F models were the Gompertz and Weibull hazard functions are considered. The QXg distribution’s Laplace transform offers an easy mathematical technique for producing analytical formulas for the QXg-F model’s unconditional survival and hazard functions. All mathematical formulas and equations necessary for analysis the QXg-F model based on Gompertz (GBH-F) and Weibull hazard-rate function (WBH-F) have been derived and analyses. Many of the new algebraic derivations have been simplified and analyzed within the framework of this paper.

Simulation analyses pointed out that the convergence properties of the MLEs were satisfied under varying censoring proportions ($$0\%,$$$$10\%,$$ $$30\%,$$ and $$50\%$$ of censoring), as expected. The proposed test statistic element formulations were developed using the QXg-F model. In addition to performing as expected, the modified chi-squared test confirmed its capacity to discover unobserved heterogeneity among small and large samples ($$n=25,$$ $$n=50,$$$$n=150,$$ $$n=350,$$ $$n=550$$and $$n=1000$$).

The simulation results shows that the proposed test for the QXg-F model performs well in both completed and censored data sets. It indicates that the test is robust as well as accurate for testing the goodness of fit of our proposed model to real-life survival data. Through the presented simulation studies, the new QXg-F model under the GBH-F and WBH-F models has proven its efficiency and flexibility, and the model’s capabilities are characterized by efficiency, adequacy, consistency, and convergence. All of these results nominate the model for statistical modeling operations for new medical data.

As for the aspect of risk analysis under the new QXg-F model, we have conducted a comprehensive study to analyze the values at risk within the framework of a set of actuarial indicators, which are used for the first time in this framework of statistical analysis. For greater robustness in the estimation and actuarial analysis processes, we used more than one method to estimate the parameters of the new model, by also presenting some important comparisons. For assessing risk under different estimation methods via simulations, the following results are highlighted:

- V-R$\left( Z;\underline{\textbf{P}}\right)$, TLV-R$\left( Z; \underline{\textbf{P}}\right)$ and TMVK$\left( Z;\underline{\textbf{P}} \right)$ increase when *q* increases for all estimation methods.
- V-R$\left( Z;\underline{\textbf{P}}\right) _{\text {WLS}}<$V-R$\left( Z; \underline{\textbf{P}}\right) _{\text {AnDE}}<$V-R$\left( Z;\underline{ \textbf{P}}\right) _{\text {MaxLE}}<$ V-R$\left( Z;\underline{\textbf{P}} \right) _{\text {OrLSE}}$ for most *q* values.
- TLV-R$\left( Z;\underline{\textbf{P}}\right) _{\text {WLS}}<$TLV-R$\left( Z;\underline{\textbf{P}}\right) _{\text {AnDE}}<$TLV-R$\left( Z; \underline{\textbf{P}}\right) _{\text {MaxLE}}<$TLV-R$\left( Z;\underline{ \textbf{P}}\right) _{\text {OrLSE}}$ for most *q* values.

For assessing risk under different estimation methods via the insurance data, the following results are highlighted:

1. For all risk assessment methods:
  $$\begin{aligned} \text {V-R}(Z;\widehat{\underline{\textbf{P}}}|1-q= & {} 50\%)<...<\text {V-R}(Z; \widehat{\underline{\textbf{P}}}|1-q=1\%), \\ \text {TLV-R}(Z;\widehat{\underline{\textbf{P}}}|1-q= & {} 50\%)<...<\text {TLV-R} (Z;\widehat{\underline{\textbf{P}}}|1-q=1\%).\\ \text {T-VC}(Z;\widehat{\underline{\textbf{P}}}|1-q= & {} 50\%)>...>\text {T-VC} (Z;\widehat{\underline{\textbf{P}}}|1-q=1\%), \\ \text {TMVK}(Z;\widehat{\underline{\textbf{P}}}|1-q= & {} 50\%)>...>\text {TMVK} (Z;\widehat{\underline{\textbf{P}}}|1-q=1\%).\\ \text {MELS}(Z;\widehat{\underline{\textbf{P}}}|1-q= & {} 50\%)>...>\text {MELS}(Z; \widehat{\underline{\textbf{P}}}|1-q=1\%). \end{aligned}$$
2. Under the all estimation method, the V-R is monotonically increasing indicator, the TLV-R in monotonically increasing indicator. However the T-VC, the TMVK and the MELS are monotonically decreasing.

Some potential points and directions for future academic works:

1. Explore further refinements and extensions of the QXg-F model. Researchers can work on developing more robust versions of the model to better capture the complexities of heterogeneity in emergency care data.
2. Investigate how time-varying covariates can be integrated into the QXg-F model. This could be important for capturing changes in patient characteristics and risk factors over time.
3. Apply the QXg-F model to real-life emergency care data and provide practical insights. This could involve analyzing specific medical conditions, patient cohorts, or healthcare facilities. See Goual et al.^39,40^ and Goual and Yousof^41^ for more relevant applications.
4. Explore other advanced validation tests and sensitivity analyses to ensure the model’s reliability and robustness, especially in the context of emergency care data where patient outcomes can be time-sensitive and complex.
5. Extend the use of the QXg-F model for predictive modeling.
6. Introduce some new risk analysis indicators under the QXg-F model.
7. Develop strategies for handling missing data within the context of emergency care data, as missing data is often prevalent in healthcare datasets. Evaluate how the model copes with missing information and propose imputation techniques.
8. Explore methods to enhance the interpretability of the QXg-F model. This is crucial for healthcare practitioners to make informed decisions based on the model’s output.
9. Investigate the feasibility of integrating the model into clinical decision support systems in emergency care settings. This could involve developing user-friendly software tools for healthcare professionals.
10. Conduct epidemiological studies using the QXg-F model to gain insights into the patterns of diseases or health conditions in specific populations, including their survival probabilities and risk factors.
11. Address ethical and privacy concerns related to the use of emergency care data, especially when conducting research involving patient records. Explore methods for de-identification and data anonymization.
12. Investigate the model’s potential to aid in resource allocation decisions in emergency care, such as optimizing the allocation of healthcare personnel and equipment based on predicted patient needs.
13. Applying the intelligent solution predictive networks and neuro-computational intelligence for analyzing the emergency care data set (see^42^ and^43^ and^44^).

## Data Availability

The dataset can be provided by Hamami Loubna upon requested.
